# Forecasting Conditional VaR of the Crude Oil Market Using GARCH-Type Models with Time-Varying High-Order Moments

**DOI:** 10.3390/e28101045

**Published:** 2026-09-22

**Authors:** Shuang Zhao, Dong Yang, Xiujiao Zhang

**Affiliations:** 1 School of Economics, Southwest Minzu University, Chengdu 610041, China; zhaoshuang@swun.edu.cn; 2 School of Statistics and Data Science, Southwestern University of Finance and Economics, Chengdu 611130, China; 224025200016@smail.swufe.edu.cn; 3 Joint Laboratory of Data Science and Business Intelligence, Southwestern University of Finance and Economics, Chengdu 611130, China

**Keywords:** conditional VaR, GARCH-type models, crude oil market, skewed generalized t distribution, time-varying high-order moments

## Abstract

Accurately modeling the distribution of the crude oil market is crucial in forecasting downside risk. In this paper, the flexible skewed generalized t (SGT) distribution is used to model the distribution of WTI and Brent crude oil market returns. The estimates of the unconditional SGT indicate that the empirical distributions of both WTI and Brent crude oil returns can be well fitted by the skewed t (ST) distribution. Furthermore, we relax the conventional GARCH-type models and allow the high-order conditional moments, such as the third and fourth moments, which are related to skewness and kurtosis, to be time-varying. The results show that all the conditional moments in the ST density function are statistically significant. Lastly, the efficiency of ST-GARCH-type models with time-varying high-order moments is evaluated based on the results from both in-sample and out-of-sample conditional value at risk (VaR) forecasting. We find that the proposed model generates superior estimates compared with the real VaR thresholds. These results have both theoretical and practical implications for financial institutions in formulating crude oil market risk management strategies and avoiding potential losses due to extreme risks in the crude oil market.

## 1. Introduction

Crude oil, as an important strategic resource, is generally valued by all countries. In recent years, the fluctuations of crude oil prices have drawn much attention from both market participants and financial practitioners; not only do fluctuations in crude oil prices have a significant impact on the development of the real economy, but they also have a certain risk impact on the financial market [1,2]. Hence, the ability to accurately forecast the risk of volatility for crude oil prices is a vital concern in both policy and financial areas [3,4,5].

Among the risk assessment tools, the value at risk (VaR) derived from the Risk Metrics Technical document [6] has proven to be an effective tool for measuring financial risk and has been widely used by financial institutions [7,8]. To improve the accuracy of VaR, a risk model should consider stylized facts, such as skewness, fat tails, and peakedness around the mean. Currently, the EWMA and GARCH-type models, based on commonly used distributions like normal, student t (T), generalized error distribution (GED), and skewed t distribution (ST), are typically treated as benchmarks in forecasting VaRs for the crude oil market [9,10,11,12].

Although the above studies consider the asymmetry and heavy-tailed nature of crude oil returns, they do not evaluate the performance of alternative distributions in terms of their ability to forecast conditional VaR thresholds. The main purpose of this study is to develop a new method to calculate conditional VaR. The approach introduced here is based on a more flexible skewed generalized t (SGT) distribution, which is proposed by Theodossiou [13] involving five parameters to fit the crude oil returns. The advantage of the SGT distribution is that many well-known distributions can be treated as special or limiting cases, such as the skewed generalized error (SGE) distribution, the generalized t (GT) distribution introduced by McDonald and Newey [14], the skewed t (ST) distribution proposed by Hansen [15], the student t (T) distribution, and the normal distribution (In Section 3.2, we will show how to obtain a specific distribution by setting particular values of the SGT distribution).

To determine which distribution is more appropriate to model the returns, we use likelihood ratio (LR) tests to choose after fitting the crude oil returns. Then, we use the proposed distribution and allow the parameters to be time-varying to capture the tail risks of the standardized return distribution. Before calculating conditional VaR, conditional mean and volatility are needed. Here, we use eight variations of GARCH-type models and allow the high-order moment parameters (e.g., conditional skewness and kurtosis) of the proposed density to be time-varying as an autoregressive process. At last, the parameters of GARCH-type models with time-varying high-order moments are obtained from the maximization of the sample log-likelihood function based on the appropriate distribution from LR tests. By using this setting, the traditional i.i.d. assumption of standardized returns is relaxed.

Our empirical analysis is based on the daily WTI and Brent spot prices. By comparing the likelihood ratio (LR) tests of five distributions with constant parameters, we find that the ST distribution with four parameters can fit the returns well. The results of maximum likelihood estimation for conditional distributions indicate that parameters in the ST distribution are statistically significant, implying that volatility, skewness, and kurtosis are time-varying. The LR tests and out-of-sample performance further reveal that ST-GARCH-type models with constant high-order moments are dominated by the same ST-GARCH-type models with time-varying high-order moments. Overall, these findings give strong evidence that the ST distribution with time-varying parameters has obvious statistical superiority for fitting WTI and Brent crude oil returns.

Finally, we use two tests, i.e., unconditional and conditional coverage tests, which are proposed by Kupiec [16] and Christoffersen [17], respectively, to assess the conditional VaR forecasting performance of the ST-GARCH-type models with time-varying high-order moments. Both in-sample and out-of-sample results indicate that ST-GARCH-type models with time-varying high-order moments proposed in this paper generate quite promising estimates of the actual VaR thresholds for WTI and Brent crude oil returns. The results further indicate that the ST-GARCH-type models with asymmetric parameters outperform the counterpart models without asymmetric parameters.

## 2. Literature Review

There are two strands of literature on the measurement of crude oil market risk; one focuses on modeling the conditional VaR based on the mean–variance framework, while the other considers the effects of conditional high-order moments (i.e., skewness and kurtosis).

Generally, most of the research on the crude oil market risks revolves around the GARCH-type models. Early studies on VaR forecasting of crude oil by Cabedo and Moya [18] show that historical simulation with an ARMA forecast approach methodology provides a flexible VaR quantification compared with the ARCH model. However, Costello et al. [19] point out that the conclusion of Cabedo and Moya [18] is mainly driven by the normal distribution assumption, and the VaR forecasts of the semi-parametric GARCH model are superior to the VaR forecasts from the ARMA with historical simulation. Kang et al. [20] investigate the performance of out-of-sample volatility forecasts of GARCH-type models for three crude oil markets and find that CGARCH and FIGARCH models are useful for forecasting the volatility of crude oil prices. Aloui and Mabrouk [21] compute the VaR of three GARCH-type models with three alternative innovation distributions for some major crude oil commodities. Their results show that considering long-range memory, fat tails, and asymmetry performs better in predicting a one-day-ahead VaR. Cheng and Hung [22] utilize the SGT distribution for describing petroleum volatilities and find that the forecasted VaR obtained using the SGT distribution provides the most accurate out-of-sample forecasts. Ghorbel and Trabelsi [23] propose a method for estimating the VaR of an energy portfolio based on the combination of models with extreme value theory. They find that GARCH-t, conditional EVT, and FIGARCH extreme value copula methods produce acceptable estimates of risk both for standard and more extreme VaR quantiles. Lyu et al. [11] use NAGARCH models with several distributions that account for asymmetry and heavy tails to forecast the VaR of crude oil markets and find that the NAGARCH model with the generalized asymmetric student-t (GAST) distribution generates more accurate VaR measurements.

Apart from the studies mentioned earlier, there are also other strands of literature that highlight crude oil market risks based on jump processes and the wavelet approach, among others. For instance, Zhang and Chen [24] and Alqahtani et al. [25] apply a GARCH model combined with a jump process to explore the dynamic dependence between changes in oil prices and stock returns. However, these studies are mostly grounded in linear regression and primarily focus on time information from a holistic perspective. On the other hand, Huang et al. [26] and Zhang and Li [27] consider the information implied in the frequency domain. This approach examines crude oil market risks from different perspectives and supplements research on periodic components in time series.

The above-mentioned existing research is of great importance for understanding and grasping the volatility characteristics and risk situation in the crude oil market. Nevertheless, the conventional GARCH-type models are still limited in the scope of the mean–variance framework. The time-varying high-order moments, i.e., the skewness and kurtosis parameters, are assumed to be constant. Since the seminal work of Hansen [15], there is substantial literature emphasizing that the high-order moments of returns are also significantly time-varying. Jondeau and Rockinger [28] use a GT distribution to model innovations in a GARCH-type model with autoregressive specification of the high-order moment parameters. They find that skewness is strongly persistent, but kurtosis is much less so for a set of daily stock indices. Bali et al. [7] extend the SGT-GARCH model and allow the conditional skewness and kurtosis to follow an autoregressive process to characterize the tail risks of the daily returns on the CRSP value-weighted index. The results indicate that their proposed models generate very accurate and robust estimates of the actual VaR thresholds. Ergün and Jun [29] use GARCH-type models with conditional skewness and kurtosis to forecast VaR for the S&P 500 stock index and find that this model provides accurate VaR forecasts. Feunou et al. [30] develop a simple procedure to evaluate parametric conditional skewness models and find that an asymmetric GARCH specification on shape parameters with a skewed generalized error distribution provides the best in-sample fit for the S&P 500 returns. Babikir et al. [31] use a GARCH(1,1) model with an error distribution assumed to be Johnson’s SU under varying skewness and kurtosis levels to model volatility for five price indices and find that models with time-varying shape parameters generate a better fit than models with constant shape parameters. Wu et al. [32] propose a realized EGARCH model with skewness and kurtosis (RealEGARCH-SK) for forecasting VaR. The empirical analysis results demonstrate that the RealEGARCH-SK model produces more accurate VaR forecasts than the traditional RealGARCH models with the normal distribution and the ST distribution. Wu and Xie [33] propose a realized EGARCH-MIDAS model with high-order moments (EGARCH-MIDAS-SK) to model volatility for five international stock market indices and find that the new model outperforms the REGARCH model in terms of in-sample fit and out-of-sample forecast performance.

In our study, we further extend the scope of flexible distribution to model the volatility of crude oil market returns by modeling the innovations using the SGT distribution. Our contribution to the literature is twofold: First, we estimate the parameters based on different distribution functions for the WTI and Brent crude oil markets. The results indicate that the characteristics of WTI and Brent crude oil returns, i.e., asymmetry and fat tails, can be well captured by the ST distribution with four parameters. Hence, we attempt to model the innovations in crude oil returns using the ST distribution and aim to improve the VaR forecasting performance. Second, we present a comparative analysis of conditional volatility equations for various ST-GARCH-type models with time-varying high-order moment parameters to model the WTI and Brent crude oil returns and use several backtesting measures to test the efficiency of the models. Our results show that the parameters of conditional volatility, skewness, and kurtosis in the ST density function are strongly significant at a common significance level. Furthermore, both in-sample and out-of-sample performance results show that the various ST-GARCH-type models with time-varying high-order moments proposed in this paper generate quite accurate and robust estimates of the actual VaR threshold.

The rest of this paper is organized as follows: Section 3 shows GARCH-type models with different volatility equations used in this paper. Then, we introduce a flexible distribution, which is called the skewed generalized t distribution, and some special and limiting cases of this distribution are discussed. Section 4 presents how to calculate the conditional VaR based on the SGT-GARCH model. Section 5 describes the data. Section 6 discusses the results and the forecasting performance of ST-GARCH-type models with time-varying high-order moments. Section 7 presents the expected shortfall (ES) calculation under the ST-GARCH-type models and reports the ES forecasting performance of the principal specifications at different coverage levels. Section 8 concludes.

## 3. GARCH-Type Models with Skewed Generalized t Distribution

### 3.1. GARCH-Type Models

Since Engle [34] and Bollerslev [35] introduced the Autoregressive Conditional Heteroscedasticity (ARCH) model and its generalized form, the GARCH model, there have been numerous extensions of this method for modeling conditional volatility. Modeling volatility has become more and more popular among financial economists. In this paper, eight of the most popular AR(1)-GARCH-type models are introduced to calculate the conditional mean and volatility (i.e., standard deviation) of crude oil market returns. Then, we use standardized returns to analyze downside risk. In general, the AR(1)-GARCH model can be written as follows:

(1) $$r_{t}=\mu_{t}+\varepsilon_{t}$$

(2) $$\mu_{t}=\alpha_{0}+\alpha_{1}r_{t-1}$$

(3) $$\varepsilon_{t}=\sigma_{t}\eta_{t}$$

(4) $$g\left(\sigma_{t}\right)=f\left(\sigma_{t-1},\eta_{t-1};\beta_{0},\beta_{1},\lambda \right)+\beta_{2}g\left(\sigma_{t-1}\right)$$

where $\mu_{t}$ and $\sigma_{t}$ represent the conditional mean and volatility for returns rt based on the available information set $F_{t-1}$ up to time t−1, respectively. $\varepsilon_{t}$ and $\eta_{t}$ denote the innovation and standardized error term at time t. $g\left(\sigma_{t-1}\right)$ represents a general form of volatility which is commonly used as $\sigma_{t-1}$ or $\sigma_{t-1}^{2}$, and λ controls asymmetry in the ARCH process. In Table 1, we summarize the conditional volatility equations in GARCH-type models that will be used in this paper.

### 3.2. Skewed Generalized t Distribution

It has been widely known that financial returns are far from normal; the standardized returns based on their conditional mean and volatility from a GARCH-type model also exhibit obvious abnormality [36]. To estimate the VaR, the common methods in the literature are (i) the variance–covariance approach (parametric method), (ii) historical simulation techniques (non-parametric method), and (iii) Monte Carlo simulation (semiparametric method). These methods only partially solve issues about the high-order moments and thus cannot fully improve the estimation accuracy of downside risk [37].

In this section, the skewed generalized *t* distribution (SGT) proposed by Theodossiou [13] is used to model the empirical distribution of crude oil returns. The SGT is quite flexible and can nest a series of known distributions and thus has been widely used in different areas [38,39,40,41].

#### 3.2.1. SGT Distribution with Constant Parameters

The probability density function of the SGT distribution can be written as follows:

(5) $$f_{SGT}\left(r;\mu ,\sigma ,\gamma ,p,q\right)=\frac{p}{2\nu \sigma q^{1/p}B\left(\frac{1}{p},q\right){\left(\frac{{\left|r-\mu +m\right|}^{p}}{q{\left(\nu \sigma \right)}^{p}{\left(\gamma \mathrm{sign}\left(r-\mu +m\right)+1\right)}^{p}}+1\right)}^{\frac{1}{p}+q}}$$

where B is the beta function, μ is the location parameter, σ>0 is the scale parameter, −1<γ<1 is the skewness parameter, and p>0 and q>0 are the parameters that control the kurtosis. m and ν are functions of the other parameters that are used to scale or shift the distribution appropriately to match the various parameterizations of the distribution.

In the original parameterization of the SGT distribution, we have

(6) $$m=\frac{2\nu \sigma \gamma q^{\frac{1}{p}}B\left(\frac{2}{p},q-\frac{1}{p}\right)}{B\left(\frac{1}{p},q\right)}$$

and

(7) $$\nu =\frac{q^{-\frac{1}{p}}}{\sqrt{\left(3\gamma^{2}+1\right)\frac{B\left(\frac{3}{p},q-\frac{2}{p}\right)}{B\left(\frac{1}{p},q\right)}-4\gamma^{2}\frac{B{\left(\frac{2}{p},q-\frac{1}{p}\right)}^{2}}{B{\left(\frac{1}{p},q\right)}^{2}}}}$$

It should be noted that m and ν generate a distribution with a mean of μ if pq>1 and a variance of $\sigma^{2}$ if pq>2. Thus, in order to take on this value for m and ν, we should guarantee pq>2.

For the high-order moments, let X be a random variable from the SGT distribution. As shown in Theodossiou [13], the *h*th moments (i.e., $E\left[{\left(X-E\left(X\right)\right)}^{h}\right]$) for pq>h are

(8) ∑r=0hhr1+γr+1+−1r1−γr+1−γh−rνσhqhpBr+1p,q−rpB2p,q−1ph−r2r−h+1B1p,qh−r+1

In particular, the skewness for pq>3 is

(9) $$\begin{array}{r} \frac{2q^{3/p}\gamma {\left(\nu \sigma \right)}^{3}}{B{\left(\frac{1}{p},q\right)}^{3}}(8\gamma^{2}B{\left(\frac{2}{p},q-\frac{1}{p}\right)}^{3}-3\left(1+3\gamma^{2}\right)B\left(\frac{1}{p},q\right)\\ \times B\left(\frac{2}{p},q-\frac{1}{p}\right)B\left(\frac{3}{p},q-\frac{2}{p}\right)+2\left(1+\gamma^{2}\right)B{\left(\frac{1}{p},q\right)}^{2}B\left(\frac{4}{p},q-\frac{3}{p}\right)) \end{array}$$

The kurtosis for pq>4 is

(10) $$\begin{array}{r} \frac{q^{4/p}{\left(\nu \sigma \right)}^{4}}{B{\left(\frac{1}{p},q\right)}^{4}}(-48\gamma^{2}B{\left(\frac{2}{p},q-\frac{1}{p}\right)}^{4}+24\gamma^{2}\left(1+3\gamma^{2}\right)B\left(\frac{1}{p},q\right)B{\left(\frac{2}{p},q-\frac{1}{p}\right)}^{2}\\ \times B\left(\frac{3}{p},q-\frac{2}{p}\right)-32\left(1+\gamma^{2}\right)B{\left(\frac{1}{p},q\right)}^{2}B\left(\frac{2}{p},q-\frac{1}{p}\right)B\left(\frac{4}{p},q-\frac{3}{p}\right)\\ +\left(1+10\gamma^{2}+5\gamma^{4}\right)B{\left(\frac{1}{p},q\right)}^{3}B\left(\frac{5}{p},q-\frac{4}{p}\right)) \end{array}$$

Then, the parameters of the SGT distribution can be estimated by maximizing the sample log-likelihood function as follows:

(11) $$\begin{array}{c} L=\sum_{t=1}^{T}\ln\left(f\left(\eta_{t}|\gamma ,p,q\right)\right) \end{array}$$

with respect to μ, σ, γ, p, q.

#### 3.2.2. Conditional SGT Distribution with Time-Varying Parameters

A body of literature has proven that the high-order moment parameters in the conditional density function exhibit time-varying characteristics [28,42,43]. There are two commonly implemented approaches that allow for time-varying high-order moments: (i) assuming the parameters of the conditional distribution, which are related to the skewness and kurtosis, to be time-varying (this is also called the autoregressive conditional density (ARCD) approach proposed by Hansen [15]), and (ii) specifying the time-varying dynamics in the high-order moments, then computing the parameters of the corresponding conditional distribution (this is also called the autoregressive conditional moment (ARCM) approach proposed by León et al. [44]).

In this paper, we adopt the ARCD approach initiated by Hansen [15] to allow the skewness and kurtosis parameters in the SGT distribution to be a function of historical information as follows:

$${\tilde{\gamma }}_{t}=\gamma_{0}+\gamma_{1}\eta_{t-1}+\gamma_{2}{\tilde{\gamma }}_{t-1}$$

$${\tilde{p}}_{t}=p_{0}+p_{1}\eta_{t-1}+p_{2}{\tilde{p}}_{t-1}$$

$${\tilde{q}}_{t}=q_{0}+q_{1}\eta_{t-1}+q_{2}{\tilde{q}}_{t-1}$$

where ${\tilde{\gamma }}_{t}$ is an unrestricted parameter which controls skewness dynamics, and ${\tilde{p}}_{t}$ and ${\tilde{q}}_{t}$ are unrestricted parameters which control kurtosis dynamics together. According to the setting of the SGT distribution, the restrictions $\left|\gamma_{t}\right|<1$, $p_{t}>0$, and $q_{t}>2$ are imposed. Here, we use the logistic transformations to satisfy the restrictions:

$$\gamma_{t}=-1+2/\left(1+\exp\left(-{\tilde{\gamma }}_{t}\right)\right)$$

$$p_{t}=exp\left({\tilde{p}}_{t}\right)$$

$$q_{t}=2+\exp\left({\tilde{q}}_{t}\right)$$

Then, the parameters of the conditional SGT-GARCH model with time-varying high-order moments can be estimated by maximizing the sample log-likelihood function

(12) $$\begin{array}{c} L=\sum_{t=1}^{T}\ln\left(f\left(\eta_{t}|\gamma_{t},p_{t},q_{t}\right)\right) \end{array}$$

with respect to the time-varying high-order moment parameters.

#### 3.2.3. Special Cases of SGT

By defining specific values or setting limitations, the SGT distribution includes several common distributions, such as the skewed generalized error distribution (SGED) of Theodossiou [45] for q=∞, the generalized t distribution (GT) of McDonald and Newey [14] for λ=0, the skewed t distribution (ST) of Hansen [15] for p=2, the skewed Laplace distribution (SLaplace) for q=∞ and p=1, the generalized error distribution (GED) of Subbotin [46] for q=∞ and λ=0, a skewed normal distribution (SNormal) for p=2 and q=∞, the student t distribution for p=2 and λ=0. Figure 1, adapted from Hansen et al. [39], gives the SGT distribution tree and shows which parameters should be set to obtain the different special cases of the SGT distribution.

## 4. Conditional VaR with SGT-GARCH Model

As for how to calculate the conditional VaR using the GARCH model with the SGT distribution, the following steps can be performed: First, we use the cumulative distribution as follows:

(13) $$\begin{array}{c} Pr\left(r_{t}\leq \theta_{t}|F_{t-1}\right)\equiv \int_{-\infty }^{\theta_{t}}f\left(r_{t}|F_{t-1}\right)dr_{t}=\phi \end{array}$$

where $r_{t}$ is the daily crude oil return, $\theta_{t}$ is the conditional threshold value, ϕ is a pre-defined coverage probability, and $f\left(r_{t}|F_{t-1}\right)$ is the conditional density of $r_{t}$.

Then Equation (13) can be rewritten using standardized returns with their conditional mean $\mu_{t}$ and standard deviation $\sigma_{t}$:

(14) $$\begin{array}{rr} Pr(r_{t} & \leq \theta_{t}|F_{t-1})=Pr\left(\frac{\left(r_{t}-\mu_{t}\right)}{\sigma_{t}}\leq \frac{\left(\theta_{t}-\mu_{t}\right)}{\sigma_{t}}|F_{t-1}\right)\\ & =Pr\left(\eta_{t}\leq a_{t}=\frac{\theta_{t}-\mu_{t}}{\sigma_{t}}\right)=\int_{-\infty }^{a_{t}}f\left(\eta_{t}\right)d\eta_{t}=\phi \end{array}$$

where $f\left(\eta_{t}\right)$ denotes the conditional density function of the SGT distribution and $a_{t}$ is the conditional threshold, which can be calculated for a given coverage probability ϕ.

Then, the conditional threshold value $a_{t}(\phi )$ is given by:

(15) $$a_{t}(\phi )=F_{SGT}^{-1}(\phi )$$

where $F_{SGT}^{-1}(\cdot )$ is the inverse of the cumulative distribution function (CDF) of the SGT distribution, defined implicitly by $\int_{-\infty }^{a_{t}}f(\eta_{t})d\eta_{t}=\phi$. Since $F_{SGT}^{-1}(\cdot )$ has no closed form in general, the threshold value is evaluated numerically.

For the traditional VaR with a normal distribution, it has been widely known that the threshold value $a_{t}$ equals −2.326 for coverage probability ϕ=1% and $a_{t}$ equals −1.645 for coverage probability ϕ=5%. However, for the conditional VaR based on the SGT-GARCH model, the threshold value $a_{t}$ is also associated with the time-varying skewness parameter $\gamma_{t}$ and kurtosis parameters $p_{t}$ and $q_{t}$. Then, the conditional threshold value $\theta_{t}$ can be calculated as follows:

(16) $$\theta_{t}=\mu_{t}+a_{t}(\phi )\sigma_{t}$$

where $\mu_{t}$ and $\sigma_{t}$ are the conditional mean and conditional standard deviation, which can be estimated using one of the GARCH-type models displayed in Section 3.

## 5. Data and Preliminary Results

In this paper, both West Texas Intermediate crude oil (WTI) prices and European Brent crude oil (Brent) prices, measured in U.S. dollars per barrel, are used to forecast the conditional VaR. We treat the observation of WTI on 20 April 2020 as an outlier since it was the first time in history that oil prices recorded negative values ($−36.98). Hence, we exclude it from our empirical analysis. The data range from January 1988 to June 2021, comprising 8439 observations for WTI and 8500 observations for Brent, respectively. The data are sourced from the U.S. Energy Information Administration (EIA).

The daily compounded return is calculated as follows:

(17) $$r_{t}=100\times \left(\ln\left(P_{t}\right)-\ln\left(P_{t-1}\right)\right)$$

where $P_{t}$ is the spot WTI or Brent crude oil price at time t. The evolution of daily prices and returns and descriptive statistics of WTI and Brent crude oil returns are provided in Figure 2 and Table 2, respectively.

From Figure 2, it is evident that both WTI and Brent crude oil markets follow a similar evolution path. Specifically, WTI (Brent) crude oil prices consistently increased until mid-2008, after which the prices sharply dropped from around $140 to about $30 per barrel within six months. Another significant price collapse can be seen in 2015, attributed to a growing supply glut. The third collapse occurred in 2020 due to the severe impact of COVID-19 on the oil industry. Notably, on 20 April 2020, the futures contract price for WTI plummeted from $19 a barrel to approximately −$37 a barrel within a matter of hours. Concurrently, WTI (Brent) returns exhibit noticeable volatility during these periods, underscoring the time-varying nature of WTI (Brent) crude oil price volatility.

Turning to Table 2, we observe that the unconditional mean and standard deviation of WTI (Brent) crude oil returns are 0.0175% (0.0174%) and 2.7972% (2.5597%), respectively. The maximum value is 42.5832% (41.2023%), while the minimum value is −72.0273% (−64.3699%). The high-order moment statistics of skewness and excess kurtosis are −1.9824 (−1.8368) and 76.1673 (69.2849), respectively. The skewness statistics are negative and significant at the 1% level, indicating that the distributions of both WTI and Brent crude oil returns are left-skewed. The excess kurtosis statistics are pretty large and significant at the 1% level, indicating that the distributions of returns exhibit leptokurtosis compared with a normal distribution. The Jarque–Bera statistic further confirms the non-normality of the returns.

Further, we calculate the standardized returns by using their conditional mean and standard deviation based on a standard AR(1)-GARCH(1,1) model:

$$r_{t}=\alpha_{0}+\alpha_{1}r_{t-1}+\sigma_{t}\eta_{t},$$

$$\sigma_{t}^{2}=\beta_{0}+\beta_{1}\sigma_{t-1}^{2}\eta_{t-1}^{2}+\beta_{2}\sigma_{t-1}^{2},$$

where $\eta_{t}$ is the standardized residual and is usually assumed to be normally distributed. Then, the descriptive statistics for the standardized residuals can be obtained.

In Table 2, the second and fourth columns present descriptive statistics of standardized residuals for WTI and Brent crude oil prices. The results indicate that the values of both the skewness and kurtosis statistics are noticeably smaller than those of raw crude oil returns. Nevertheless, these values are still statistically large and significant at the 1% level, suggesting that the standard AR(1)-GARCH(1,1) model cannot fully capture the high-order moment characteristics. The Jarque–Bera statistics further indicate that the null hypothesis of normality for the standardized residuals should be rejected.

Table 3 presents the estimation results for different unconditional distribution functions with constant parameters for both WTI and Brent. Among the distributions considered—the SGT distribution, the GT distribution, the ST distribution, the symmetric T distribution, and the normal distribution—the normal distribution exhibits the largest likelihood ratio (LR) value. This suggests that the empirical distribution of WTI (Brent) crude oil returns significantly deviates from normality. The skewness parameters (γ) for the SGT and ST distributions are both around −0.05 (−0.03) with corresponding t statistics of approximately −3.20 (−2.40), indicating left-skewed empirical distributions for crude oil returns. In the case of the SGT distribution, both the skewness and the two kurtosis parameters are statistically significant. The LR statistics strongly reject the generalized t, symmetric t, and the normal distribution. However, the LR statistic does not reject the null hypothesis that the SGT and ST distributions provide equally good fits, suggesting that the ST distribution effectively captures the empirical return distributions of WTI and Brent crude oil markets. Furthermore, the estimates of the kurtosis parameter *p* under the SGT distribution are 2.0458 (0.11) for WTI and 1.8482 (0.09) for Brent, corresponding to *t* statistics of 0.42 and −1.69, respectively. Since *p* does not deviate significantly from 2, imposing the restriction p=2, which yields the ST distribution, does not sacrifice explanatory power (the *t* statistics of the kurtosis parameter *p* are $\left(2.0458-2\right)/0.11\approx 0.42$ for WTI and $\left(1.8482-2\right)/0.09\approx -1.69$ for Brent, respectively). This confirms that the ST distribution sufficiently fits the empirical distribution of WTI and Brent crude oil returns. Consequently, in the subsequent sections, we exclusively employ the ST distribution to characterize the tail risk of the standardized return distribution.

## 6. The Performance of Conditional VaR Based on ST-GARCH-Type Models

In this section, we first discuss the results of the ST-GARCH-type models with constant and time-varying high-order moment parameters, and then we show that modeling the empirical distribution of WTI (Brent) crude oil returns using the conditional ST density function generates a promising result compared with the actual VaR thresholds.

### 6.1. Estimation Results

Table 4 displays the estimation results for WTI and Brent returns using standard GARCH models based on the ST distribution with constant high-order moment parameters. The AR(1) coefficients $\alpha_{1}$ in the conditional mean equations for WTI and Brent returns exhibit opposing dynamic dependencies in the daily WTI (Brent) crude oil prices. Specifically, the AR(1) coefficients for WTI (Brent) range between −0.0277 (0.0265) and −0.0227 (0.0315), all of which are statistically significant at least at the 5% level. In the conditional variance equations, all parameters are also statistically significant at the 1% level, indicating significant time variation in the volatility equations of WTI (Brent) crude oil returns. The skewness parameter λs in the conditional volatility equations of Table 4 is highly significant at the 1% level, revealing the presence of asymmetric volatility responses to historical positive and negative shocks. Log-likelihood values and information criteria such as AIC and BIC for ST-GARCH-type models further validate that models with asymmetric parameters outperform their counterparts without such parameters in most cases. Based on AIC and BIC results, EGARCH for WTI and Brent exhibits the lowest AIC and BIC values, indicating that this model provides the best in-sample performance among the considered models.

Additionally, from Table 4, it is evident that both skewness (γ) and kurtosis (q) parameters are statistically significant at the 5% level. Specifically, the γ parameters remain negative. In contrast, the q parameters are relatively more minor (i.e., qs are not equal to ∞), suggesting that standardized WTI (Brent) crude oil returns are left-skewed and leptokurtic.

A comparative analysis shows that ST-GARCH models with time-varying high-order moments offer a superior fit for both WTI and Brent crude oil returns compared to ST-GARCH models with constant high-order moments. In Table 5 and Table 6, estimates of $\gamma_{1}$ and $\gamma_{2}$, which control the time-variation of conditional skewness $\gamma_{t}$, are all statistically significant at the 1% level, except for $\gamma_{1}$ in the GARCH, AGARCH, and TGARCH models, where significance is observed at the 10% level. For the ST-GARCH-type models with time-varying high-order moments, the optimizer employs the heuristic optimization method differential evolution, proposed by Storn and Price [47], to search for parameters that minimize a highly nonlinear objective function. Moreover, the skewness parameter ${\tilde{\gamma }}_{t}$ exhibits a certain degree of persistence, with $\gamma_{2}$ ranging between 0.2039 and 0.4833.

Similarly, estimates of the kurtosis parameters $q_{1}$ and $q_{2}$ are significant at the 5% level across various specifications, except for $q_{1}$ in the TS-GARCH model, which retains significance at the 10% level. Correspondingly, the kurtosis parameter ${\tilde{q}}_{t}$ displays persistence, varying between 0.0658 and 0.2795 in relation to $q_{2}$. However, it is worth noting that the persistence of the kurtosis parameter ${\tilde{q}}_{t}$ is not as pronounced as that of the skewness parameter ${\tilde{\gamma }}_{t}$.

Furthermore, compared to ST-GARCH models with constant high-order moments, likelihood values across different specifications substantially decrease for ST-GARCH models with time-varying high-order moment parameters. This sharp decline in likelihood values suggests that the ST-GARCH models with time-varying high-order moments provide a significantly improved goodness of fit.

Figure 3 illustrates the time series of conditional moments for WTI and Brent crude oil returns from the ST-EGARCH model with time-varying high-order moment parameters. (For simplicity, we present the ST-EGARCH model results with time-varying high-order moments. The results for other specifications exhibit similar patterns). Skewness and kurtosis are computed using Equations (9) and (10), respectively. The graph demonstrates the presence of time variation in conditional moments for both WTI and Brent crude oil returns. Although conditional volatility and kurtosis for WTI and Brent crude oil returns exhibit similar dynamics, there are notable distinctions in the patterns of conditional skewness. Specifically, when compared with the Brent crude oil market, the skewness of the WTI crude oil market indicates a higher probability of leaning towards the negative in the financial system. This trend may result in increased financial risks or losses for participants in the crude oil market. We have also calculated the proportions of negative skewness frequency for WTI and Brent crude oil returns, with values of 0.97 for WTI and 0.62 for Brent, respectively. These figures reinforce the notion that the WTI crude oil market is more susceptible to extreme risks or losses compared to the Brent crude oil market. This observation is also supported by the findings of Bin et al. [48] and Okoroafor and Leirvik [49].

In addition, we calculate descriptive statistics for conditional skewness and kurtosis. The average conditional skewness for WTI (Brent) is −1.80 (−5.60), accompanied by a relatively large standard deviation of 32.85 (35.85). Regarding conditional kurtosis, the average value is 119.37 (87.31), with a standard deviation of 364.56 (268.19). Furthermore, we compute the AR(1) coefficients for conditional skewness and kurtosis, yielding 0.3449 (−0.1666) and 0.8848 (0.8754), respectively. These coefficients are statistically significant at the 1% level, implying substantial autocorrelation in conditional high-order moments.

### 6.2. Evaluation of In-Sample Conditional VaR with ST-GARCH-Type Specifications

Table 7 presents statistics regarding the VaR thresholds $\left(a_{t}\right)$ for various ST-GARCH specifications with coverage probabilities $\left(\phi \right)$ at 0.5%, 1%, 2.5%, 5%, and 10%, based on the entire sample. In Table 7, columns 2–9 provide statistics for conditional VaR using eight ST-GARCH-type models. The first row displays the average estimated thresholds, while the second row presents both the actual and expected (in parentheses) counts of standardized returns falling below each threshold. The third row introduces two tests for assessing whether the observed and expected counts of observations below each threshold are statistically the same. The first test is the unconditional test proposed by Kupiec [16], which is formulated as follows:

(18) $$LR^{uc}=-2ln\left[{\left(1-p_{0}\right)}^{N-y}p_{0}^{y}\right]+2ln\left[{\left(1-\left(y/N\right)\right)}^{N-y}{\left(y/N\right)}^{y}\right]$$

where $p_{0}$ is the nominal tail probability, and p is the empirical tail probability. N is the number of sample observations and y is the number of observations that fall below the threshold $a_{t}$. The $LR^{uc}$ statistic follows a chi-square distribution with one degree of freedom, $\chi^{2}\left(1\right)$.

However, $LR^{uc}$ given in Equation (18) simply counts violations through the whole period and does not consider the presence of volatility clustering. Thus, it may have incorrect conditional coverage [7]. In order to avoid the inaccurate VaR estimates of the unconditional test, Christoffersen [17] develops a likelihood ratio test of the null hypothesis of serial independence against the alternative of first-order Markov dependence.

The indicator variable $I_{t}$ is constructed as

(19) $$I_{t}=1(r_{t}<\theta_{t}),\;\theta_{t}=-{VaR}_{t}$$

Then, let $N_{ij}$ denote the number of observations on which j occurs given that i occurred on the previous day; n is the number of exceptions, π=n/N, $\pi_{1}=N_{11}/n$, and $\pi_{0}=N_{01}/\left(N-n\right)$. The independence likelihood ratio statistic is

(20) $$\begin{array}{c} LR^{ind}=-2ln\left[{\left(1-\pi \right)}^{\left(N_{00}+N_{10}\right)}\pi^{\left(N_{01}+N_{11}\right)}\right]+2ln\left[{\left(1-\pi_{0}\right)}^{N_{00}}\pi_{0}^{N_{01}}{\left(1-\pi_{1}\right)}^{N_{10}}\pi_{1}^{N_{11}}\right] \end{array}$$

which is asymptotically chi-squared with one degree of freedom under the null hypothesis. Then the conditional coverage test is given by

(21) $$\begin{array}{c} LR^{cc}= \end{array}LR^{uc}+LR^{ind}$$

where the $LR^{cc}$ test statistic follows an asymptotic chi-square distribution with two degrees of freedom, $\chi^{2}\left(2\right)$.

As indicated in Table 7 and Table 8, the unconditional coverage test statistics do not provide sufficient evidence to reject the null hypothesis, suggesting that the expected and actual counts of observations falling below the estimates at various quantiles are statistically similar across most specifications. Thus, for both WTI and Brent crude oil returns, ST-GARCH-type models yield highly accurate VaR estimates at the specified coverage probabilities. However, there are some exceptions, notably the VaR estimates for the ST-GARCH model at the 1%, 2.5%, and 5% levels; the ST-EGARCH model at the 1% and 2.5% levels; the ST-NAGARCH model at the 0.5%, 1%, and 2.5% levels; the ST-IGARCH model at the 5% level; and the ST-TSGARCH model at the 1% and 5% levels.

Moreover, both Table 7 and Table 8 present the results of conditional coverage tests for both WTI and Brent crude oil returns. These results reveal that, for most coverage probabilities, the conditional coverage test does not yield enough evidence to reject the null hypothesis. However, exceptions exist in the cases of the ST-GARCH, ST-TGARCH, and ST-TSGARCH models, which exhibit partial statistical significance at the 1%, 2.5%, and 5% levels. This observation suggests that the in-sample VaR estimates at these levels are influenced by autocorrelation.

### 6.3. Evaluation of Out-of-Sample Conditional VaR with ST-GARCH-Type Specifications

Further, we give the out-of-sample conditional VaR performance of ST-GARCH-type models with time-varying high-order moments using the aforementioned tests for both WTI and Brent crude oil returns. Here, an out-of-sample approach based on the rolling-window method is utilized to forecast the VaR estimates with different specifications. This is more reasonable because risk managers usually estimate the VaR on the basis of historical information and then use these estimates to evaluate downside risks. The detailed procedure is listed as follows:

First, a 10-year rolling sample, i.e., from 4 January 1988 to 31 December 1997, is utilized to estimate the conditional VaR with ST-GARCH-type models. Then we keep the estimates unchanged in the next year, 1998, to evaluate the performance (in Appendix A, we summarize the holdout sample sizes for both WTI and Brent crude oil returns). Second, the estimated VaR (based on the rolling sample) is used to compute the number of returns falling in the 0.5%, 1%, 2.5%, 5%, and 10% quantiles of each estimated probability distribution in the 1998 holdout sample. Third, the estimated sample moves forward by deleting the sample in 1988 and thus starting from 1989. The new estimated VaR is used to calculate the number of returns falling in the same quantiles based on the new holdout sample. The rolling process will continue until the estimated samples are used up. At last, 24 years of holdout samples in total are produced.

Table 9 and Table 10 further confirm the strong performance of our conditional VaR models. Irrespective of the chosen GARCH model, the actual and expected counts are closely aligned across most coverage probabilities for both WTI and Brent crude oil returns. Specifically, concerning WTI crude oil returns, the conditional coverage test fails to reject the null hypothesis for most coverage probabilities, except the GARCH model for 2.5%, the EGARCH model for 1%, the AGARCH model for 0.5%, and the TS-GARCH model for 0.5%. For Brent crude oil returns, the conditional coverage test does not provide enough evidence to reject the null hypothesis, except for the GARCH model at 1% and 2.5%, the NAGARCH model at 2.5% and 5%, the AGARCH model at 5% and 10%, the IGARCH model at 5%, and the TS-GARCH model at 2.5%. Overall, the results of both unconditional and conditional coverage tests underscore the robustness and accuracy of the estimated VaR thresholds. In Appendix B, we present the results of GARCH-type models without time-varying skewness and kurtosis for both WTI and Brent crude oil returns. According to the $LR^{uc}$ statistics, all the GARCH-type models generate inaccurate conditional VaR estimates for the 0.5%, 1%, 2.5%, 5%, and 10% levels, both in-sample and out-of-sample, except the normal GARCH models, which exhibit appropriate VaR values for the 2.5% and 5% loss probability levels. The findings from the normal GARCH models indicate that the process controlling the tail characteristics will change as we move further out in the tail. Bali et al. [7] and Engle and Manganelli [50] find similar results.

### 6.4. Comparison with Historical Simulation

As discussed in Section 3.2, VaR can be calculated using three different approaches: the variance–covariance approach, historical simulation, and Monte Carlo simulation. Among them, historical simulation is relatively easy to implement and can therefore serve as a benchmark. In this section, an out-of-sample analysis based on the same rolling-window method is implemented to forecast VaR estimates using the historical simulation approach.

Table 11 shows that the historical simulation method consistently produces more exceedances than expected for both WTI and Brent crude oil returns across all coverage probabilities. Furthermore, both the unconditional coverage test and the conditional coverage test reject the null hypothesis at the 5% significance level. These results indicate that the historical simulation method fails to capture the higher-order moment risks of crude oil markets, which leads to an underestimation of downside risk.

### 6.5. Comparison with GARCH-Type Models with Alternative Distributions

The preceding analysis has demonstrated the potential benefits of leveraging time-varying high-order moments within ST-GARCH-type models. However, the distributions examined thus far, originating from the SGT distribution, cannot encompass all alternative distributions like Pearson’s Type IV distribution [51] and Johnson’s SU distribution [52]. Consequently, an outstanding question that remains to be answered by the previous analysis pertains to whether ST-GARCH-type models with time-varying high-order moments consistently outperform GARCH-type models based on alternative distributions regarding out-of-sample conditional VaR performance (For the sake of clarity, the methodological details of the conditional Pearson’s Type IV and Johnson’s SU distributions with time-varying parameters are provided in Appendix C).

To address this question, we propose an assumption wherein WTI and Brent crude oil returns adhere to an AR(1)-GARCH(1,1) model, with standardized residuals following Pearson’s Type IV and Johnson’s SU distributions with time-varying high-order moments, respectively. Although Pearson’s Type IV distribution is only one distribution in the Pearson system of distributions, the other types cannot effectively capture the pronounced fat-tail characteristics in returns. Hence, we solely consider Pearson’s Type IV distribution for this analysis. In accordance with the quantitative criteria set forth by the Basel Committee, we evaluate the out-of-sample conditional VaR performance at a 99% confidence level, employing both unconditional and conditional coverage test statistics. The results are illustrated in Figure 4. For the sake of clarity, we present the results from the ST-EGARCHSK model. The outcomes from the other ST-GARCHSK models are analogous and can be provided upon request.

Several intuitive conclusions can be drawn from Figure 4. Notably, during periods of high volatility in crude oil markets, VaRs for these models experience significant reductions. However, distinct distribution assumptions yield noticeable disparities in VaR forecasts. For instance, the ST-EGARCH and JohnsonSU-EGARCH models generate comparable VaRs, while the EGARCH model employing the PearsonIV distribution tends to exhibit a more aggressive stance, leading to less accurate VaR predictions. These findings reaffirm that ST-GARCH-type models featuring time-varying high-order moments offer remarkably accurate and resilient estimates of actual VaR thresholds. In Appendix D, we present the results for GARCH-type models whose standardized residuals follow Pearson’s Type IV distribution and Johnson’s SU distribution for both WTI and Brent crude oil returns. According to the LR^uc^ and LR^cc^ statistics, neither of these two distributions can dominate the ST distribution.

### 6.6. Comparison with ST-GARCH-Type Models with Time-Varying Skewness or Kurtosis Parameters

The previous analysis has shown that ST-GARCH-type models would benefit from employing time-varying high-order moments, as opposed to constant estimates. On the other hand, an outstanding question that remains unsolved by the previous analysis is the marginal contribution of time-varying skewness and kurtosis with respect to solely imposing constant restrictions on the high-order moments. To answer this question, we consider two restricted variants, one with time-varying skewness and constant kurtosis and another with constant skewness and time-varying kurtosis, and compare them with the current specification under the same out-of-sample protocol. Table 12 presents the out-of-sample conditional VaR performance of ST-GARCH-type models with time-varying skewness or kurtosis parameters for WTI and Brent crude oil returns. For the sake of clarity, we present two types of models: one is the ST-GARCH model, and one is the ST-EGARCH model, which considers the asymmetric effects of shocks.

A comparison of the results for models incorporating time-varying skewness or kurtosis reveals that these two features play markedly different roles in conditional VaR forecasting. For the WTI market, GARCH and EGARCH specifications with time-varying skewness and constant kurtosis are uniformly dominated by their counterparts with constant skewness and time-varying kurtosis across all coverage probabilities. This finding indicates that the time-varying nature of kurtosis is more important than that of skewness for forecasting conditional VaR. Analogous results emerge for the Brent market, where time-varying kurtosis again proves more important than time-varying skewness. Nevertheless, when skewness and kurtosis are both allowed to vary over time, the conditional VaR forecasting performance improves further, as documented in Table 9 and Table 10.

### 6.7. The Stability of the ST Distribution for WTI and Brent Crude Oil Returns

As discussed in Section 3.2.3, an ST distribution is a special case within the broader SGT distribution when the kurtosis parameter p is equal to 2. Therefore, it is crucial to investigate the stability of the ST distribution across varying sampling times. In this section, we undertake the task of estimating the parameters of SGT distributions using the rolling sample framework introduced in Section 6.3. The primary objective is to discern any temporal shifts within the distribution dynamics. Furthermore, we calculate the p-values for the hypothesis that the kurtosis parameters are equal to 2 across different years. The outcomes of this analysis are illustrated in Figure 5.

Figure 5 shows p values of kurtosis parameters for both WTI and Brent crude oil returns based on the rolling sample. The blue horizontal lines denote a 5% significance level. The results indicate that the p values are higher than the 5% significance level most of the time except in 2019 for WTI and 2017 for Brent, indicating no significant differences between SGT and ST distributions. Thus, the ST distribution can capture the characteristic of high-order moments for both WTI and Brent crude oil returns well.

## 7. The Performance of Expected Shortfall Based on ST-GARCH-Type Models

This paper focuses on the calculation of VaR based on ST-GARCH-type models with time-varying skewness and kurtosis parameters. However, the framework can be extended straightforwardly to expected shortfall (ES), which is a coherent and well-defined risk measure in the general case. In this section, we present the ES calculation under ST-GARCH-type models and then report the ES forecasting performance of the principal specifications at different coverage levels.

### 7.1. ES with ST-GARCH-Type Models and Evaluation Method

By definition, ES at level ϕ is the average loss in the worst ϕ fraction of cases. As shown in Section 4, the conditional VaR threshold is $\theta_{t}=\mu_{t}+a_{t}(\phi )\sigma_{t}$ in Equation (16), where $a_{t}(\phi )$ is the ϕ-quantile of the standardized innovation distribution, evaluated as discussed in Section 4.

Analogously, the standardized ES multiplier, the conditional tail expectation of the standardized SGT distribution, evaluated at the same quantile $a_{t}(\phi )$ that the VaR uses, is given by

(22) $$\begin{array}{c} e_{t}(\phi ) \end{array}=\frac{1}{\phi }\int_{-\infty }^{a_{t}(\phi )}xf(x)dx$$

and the conditional ES threshold is

(23) $$\begin{array}{c} \theta_{t}^{ES}=\mu_{t}+e_{t}(\phi )\sigma_{t}=\mu_{t}+\frac{\sigma_{t}}{\phi }\int_{-\infty }^{a_{t}(\phi )}xf(x)dx \end{array}$$

where f(·) is the ST probability density function, and $\mu_{t}$ and $\sigma_{t}$ are the conditional mean and conditional standard deviation estimated using one of the GARCH-type models presented in Section 3. These equations are used to compute the 1-day ES thresholds and to evaluate the performance of ES under the ST-GARCH-type models.

To evaluate the performance of ES with ST-GARCH-type models, we employ the ES test of McNeil and Frey [53], a bootstrap-based backtesting method for assessing the accuracy of ES and VaR forecasting models in risk management (Since the null distribution of the McNeil and Frey [53] test statistic is unknown, *p*-values are obtained via bootstrap to mitigate any bias. The detailed procedure is given in [53]). The null hypothesis is that the excess conditional shortfall (the excess of the actual series over VaR when a violation occurs) is i.i.d. and has zero mean.

### 7.2. Evaluation of Out-of-Sample ES Thresholds with ST-GARCH-Type Specifications

Without loss of generality, Table 13 reports the out-of-sample ES thresholds, with the corresponding *p*-values of the ES tests of McNeil and Frey [53] in parentheses, at the 0.5%, 1%, and 5% levels for WTI and Brent crude oil returns, based on GARCH and EGARCH models with the ST distribution. The results clearly indicate that the ES thresholds are smaller than the VaR thresholds under the same specifications. Additionally, the *p*-values of the ES tests all exceed the 10% significance level, indicating that the ES forecasts are accurately calibrated.

## 8. Conclusions

In recent years, the crude oil market has experienced heightened levels of extreme volatility, surpassing historical levels. It has been observed that traditional GARCH-type models are inadequate in capturing the phenomenon of leptokurtosis in returns. In this study, we commence by employing the skewed generalized t distribution (SGT) with various specifications to model the unconditional distribution of daily returns of WTI and Brent crude oil prices, spanning from 4 January 1988 to 30 June 2021. The findings demonstrate that the skewed t distribution (ST) effectively captures the tail characteristics of the empirical WTI and Brent return distributions, particularly when p=2. To extend the Bollerslev [35] GARCH model, we incorporate time-varying skewness and kurtosis parameters within the ST density function. This expansion relaxes the assumption of independent and identically distributed standardized returns. Our maximum likelihood estimates highlight the substantial significance of the skewness and kurtosis parameters within the ST density function. Additionally, the results of likelihood ratio tests underscore the superiority of the ST distribution over GT, T, and normal distributions. Furthermore, our analysis reveals that ST-GARCH-type models with asymmetric parameters exhibit enhanced performance compared to their counterparts lacking asymmetric parameters.

Lastly, we delve into the role of time-varying high-order moments in forecasting conditional VaR. We employ two evaluation tests, namely the unconditional coverage test and the conditional coverage test, to assess the in-sample and out-of-sample forecasting performance of conditional VaR. Moreover, we subject our model to a battery of tests to ensure its robustness. Collectively, our findings demonstrate that ST-GARCH-type models with time-varying high-order moments deliver superior forecasting performance for risks in both the WTI and Brent crude oil markets.

This research serves as a valuable theoretical reference and practical empirical approach for policymakers, financial institutions, and investors seeking to formulate effective risk management strategies within the crude oil market. It offers insights into mitigating potential losses resulting from extreme risks in this domain. Additionally, accurate VaR estimates are imperative in portfolio selection studies. Hence, this paper presents a novel risk assessment tool for portfolio management in the realm of crude oil products.

## Figures and Tables

**Figure 1:**
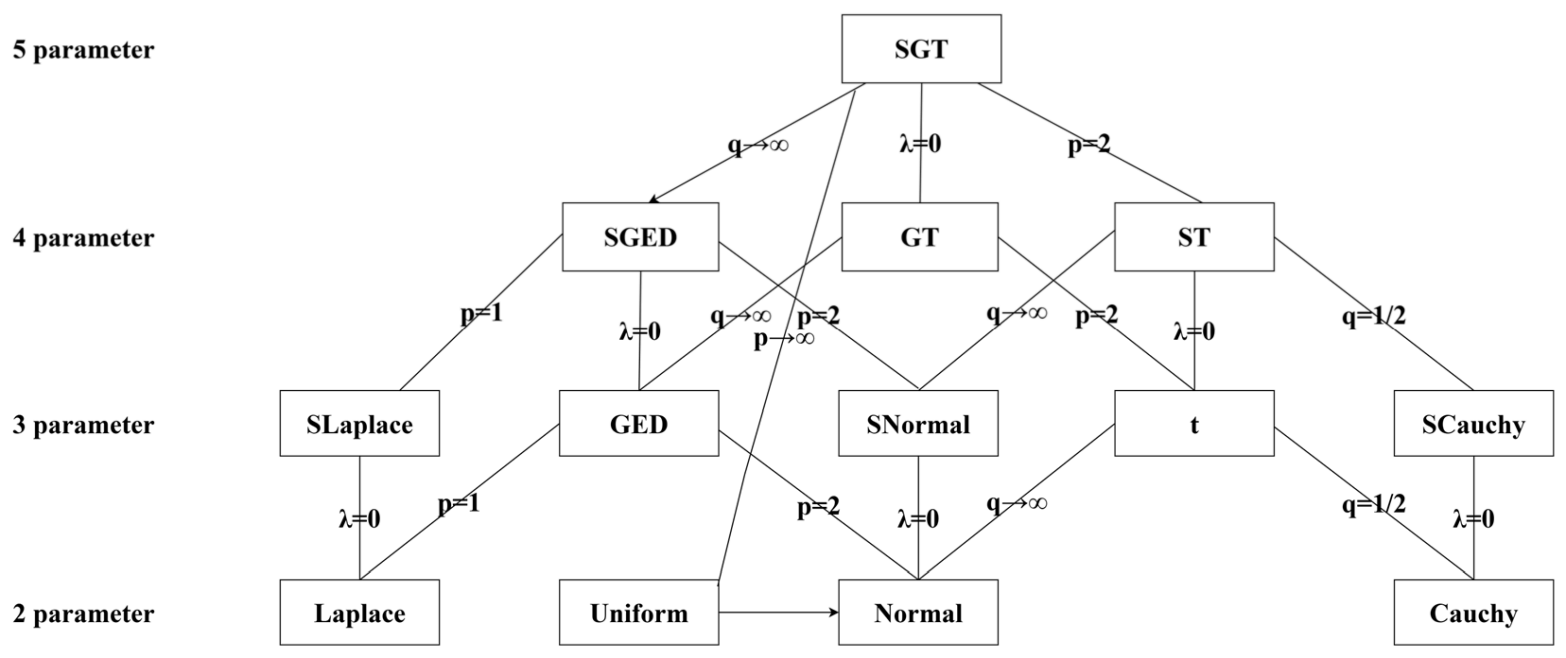
The skewed generalized *t* distribution tree.

**Figure 2:**
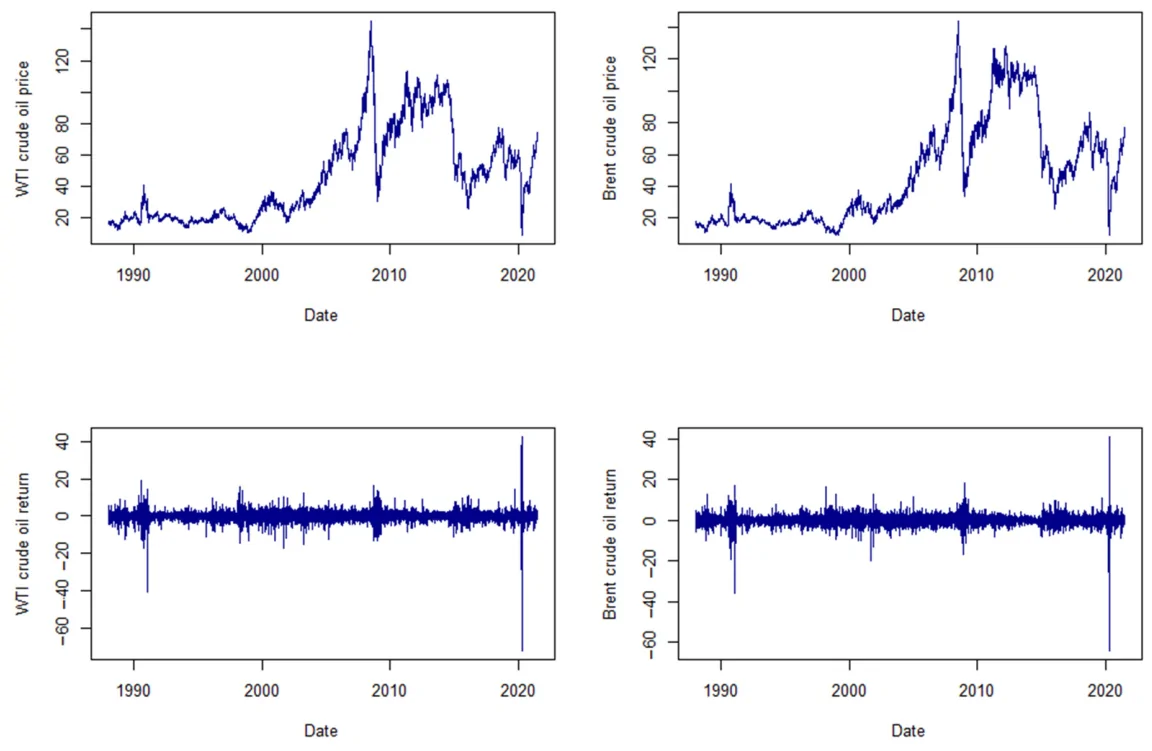
Daily WTI and Brent crude oil prices and returns.

**Figure 3:**
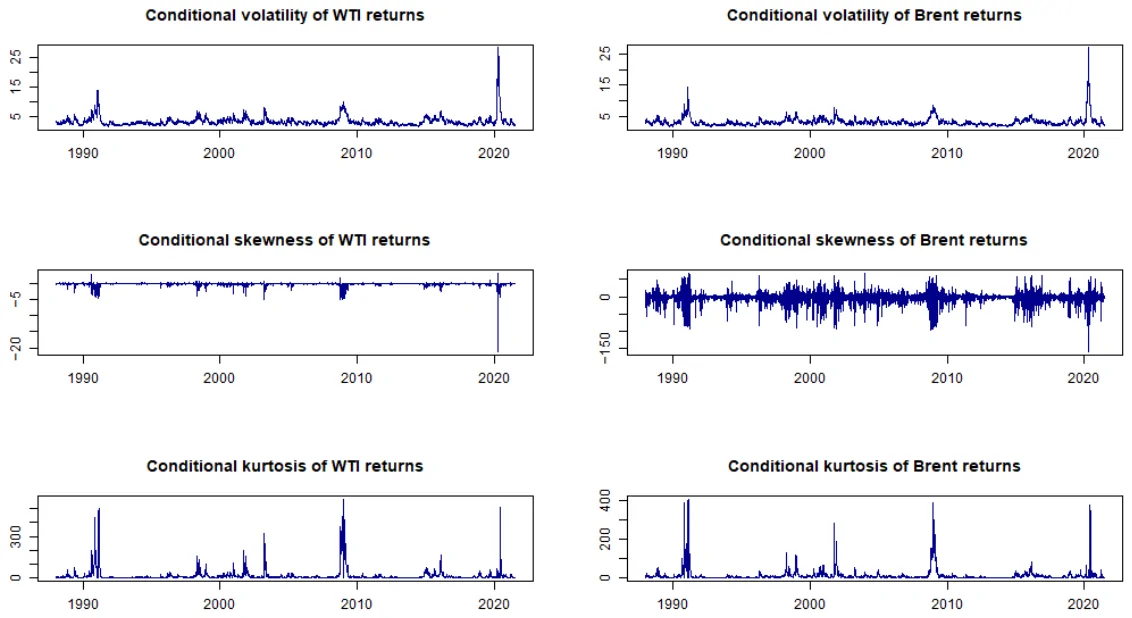
Conditional moments of WTI and Brent crude oil returns.

**Figure 4:**
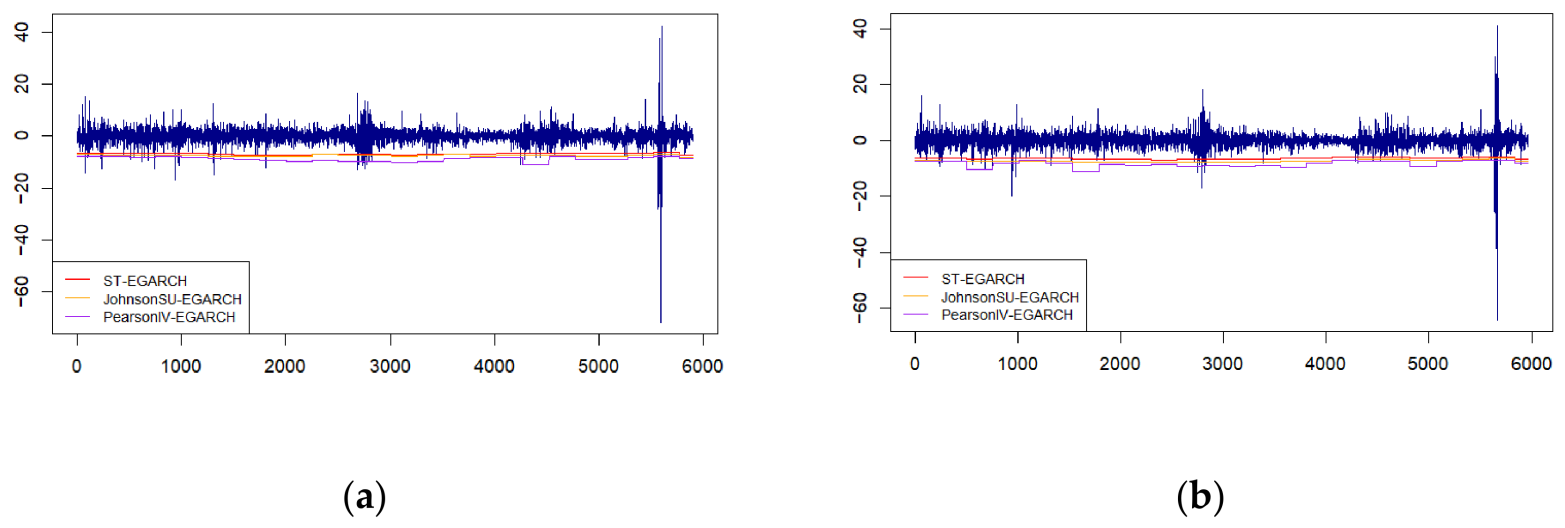
(**a**,**b**) show the 1% VaR forecast values of WTI and Brent crude oil returns, respectively.

**Figure 5:**
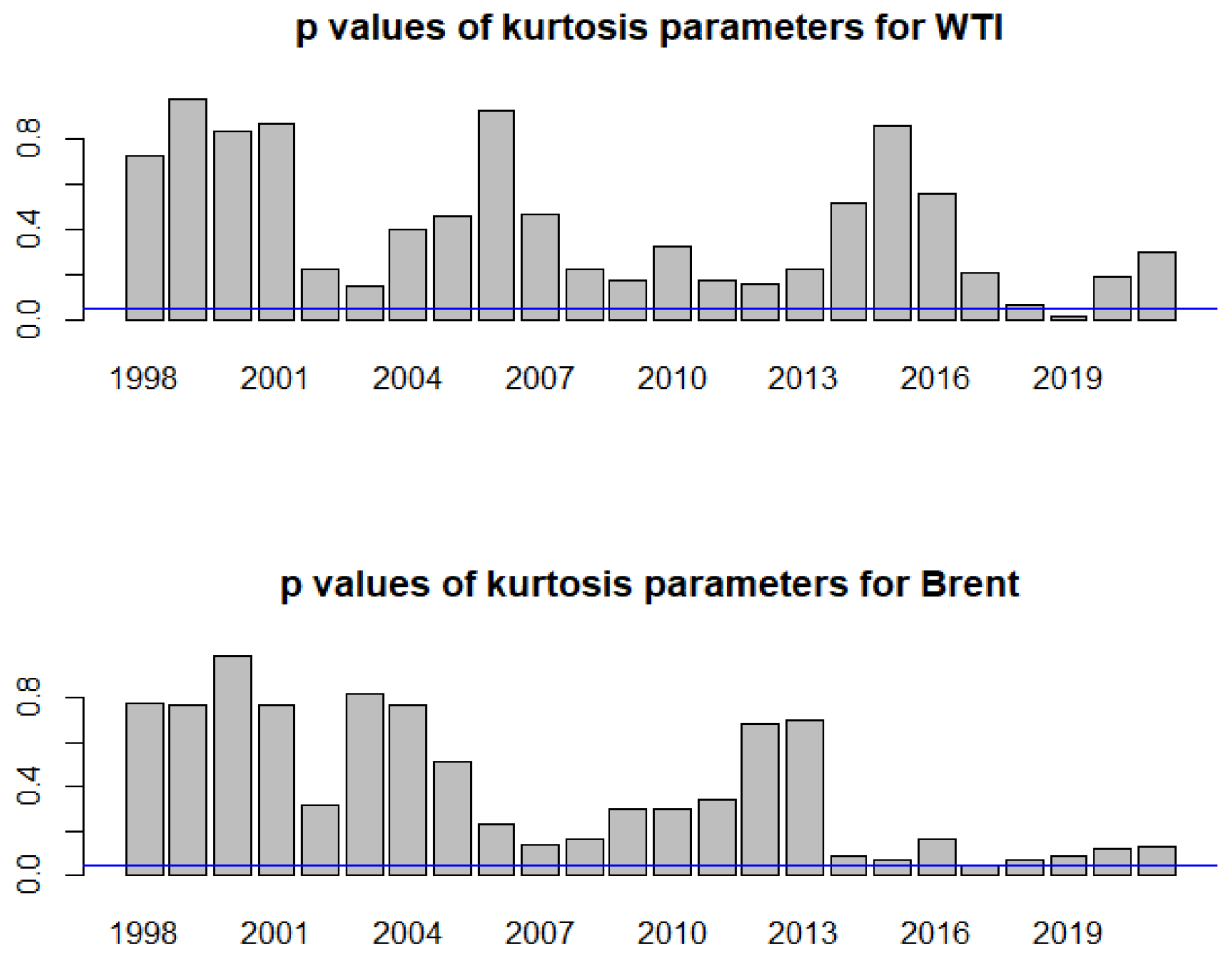
*p* values of SGT distributions for WTI and Brent crude oil returns. Notes. The blue horizontal lines denote a 5% significance level.

**Table 1:** Alternative conditional volatility equations.

Model	Formulations	Conditions
GARCH	$\sigma_{t}^{2}=\beta_{0}+\beta_{1}\sigma_{t-1}^{2}\eta_{t-1}^{2}+\beta_{2}\sigma_{t-1}^{2}$	$\beta_{0}>0,\beta_{1},\beta_{2}\geq 0$ $\beta_{1}+\beta_{2}<1$
Exponential GARCH	$\ln\sigma_{t}^{2}=\beta_{0}+\beta_{1}\left[\left|\eta_{t-1}\right|-E\left(\left|\eta_{t-1}\right|\right)\right]+\beta_{2}\ln\sigma_{t-1}^{2}+\lambda \eta_{t-1}$	no parameter restrictions
Nonlinear asymmetric GARCH	$\sigma_{t}^{2}=\beta_{0}+\beta_{1}\sigma_{t-1}^{2}{\left(\lambda +\eta_{t-1}\right)}^{2}+\beta_{2}\sigma_{t-1}^{2}$	$\beta_{0}>0,\beta_{1},\beta_{2}\geq 0$ $\beta_{1}\left(1+\lambda^{2}\right)+\beta_{2}<1$
Asymmetric GARCH	$\sigma_{t}^{2}=\beta_{0}+\beta_{1}{\left(\lambda +\sigma_{t-1}\eta_{t-1}\right)}^{2}+\beta_{2}\sigma_{t-1}^{2}$	$\beta_{0}>0,\beta_{1},\beta_{2}\geq 0$ $\beta_{1}+\beta_{2}<1$
GJR-GARCH	$\sigma_{t}^{2}=\beta_{0}+\beta_{1}\sigma_{t-1}^{2}\eta_{t-1}^{2}+\beta_{2}\sigma_{t-1}^{2}+\lambda I_{t-1}\sigma_{t-1}^{2}$ $I_{t-1}=1$ *for* $\sigma_{t-1}\eta_{t-1}<0$ *and* $I_{t-1}=0$ *otherwise*	$\beta_{0}>0,\beta_{1},\beta_{2}\geq 0$ $\beta_{1}+\beta_{2}<1$
Integrated GARCH	$\sigma_{t}^{2}=\beta_{0}+\left(1-\beta_{2}\right)\sigma_{t-1}^{2}\eta_{t-1}^{2}+\beta_{2}\sigma_{t-1}^{2}$	$\beta_{0}>0,\beta_{2}\geq 0$
Threshold GARCH	$\sigma_{t}=\beta_{0}+\beta_{1}\sigma_{t-1}\left|\eta_{t-1}\right|+\beta_{2}\sigma_{t-1}+\lambda I_{t-1}\sigma_{t-1}\eta_{t-1}$ $I_{t-1}=1$ *for* $\sigma_{t-1}\eta_{t-1}<0$ *and* $I_{t-1}=0$ *otherwise*	$\beta_{0}>0,\beta_{1},\beta_{2}\geq 0$ $\beta_{1}+\beta_{2}<1$
TS-GARCH	$\sigma_{t}=\beta_{0}+\beta_{1}\sigma_{t-1}\left|\eta_{t-1}\right|+\beta_{2}\sigma_{t-1}$	$\beta_{0}>0,\beta_{1},\beta_{2}\geq 0$ $\beta_{1}+\beta_{2}<1$

Notes. The parameter λ in the conditional volatility equations above reflects an asymmetric response to past shocks. For convenience, all formulations are written as GARCH(1,1) forms.

**Table 2:** Descriptive statistics for WTI and Brent crude oil returns.

Statistics	WTI Return	WTI Standardized Residual	Brent Return	Brent Standardized Residual
Mean	0.0175	−0.0027	0.0174	0.0000
Median	0.0747	0.0256	0.0364	0.0162
Maximum	42.5832	6.4854	41.2023	5.0133
Minimum	−72.0273	−8.9832	−64.3699	−6.3464
Std. Dev.	2.7972	1.0000	2.5597	1.0001
Skewness	−1.9824 (0.0267) ***	−0.3955 (0.0267)	−1.8368 (0.0266) ***	−0.1591 (0.0266) ***
Excess kurtosis	76.1673 (0.0533) ***	3.5480 (0.0533) ***	69.2849 (0.0531) ***	2.0019 (0.0531) ***
Jarque-Bera	2,045,465.3682	4646.3217	1,704,919.5023	1455.1970

Notes. This table shows the descriptive statistics of the daily percentage returns and standardized residuals of the WTI and Brent crude oil prices. The sample period ranges from 4 January 1988 to 30 June 2021 (8439 daily observations for WTI and 8500 daily observations for Brent, respectively). The standardized residual is calculated by using MLE of the following setting: $\begin{array}{cc} r_{t} & =\alpha_{0}+\alpha_{1}r_{t-1}+\sigma_{t}\eta_{t}\\ \sigma_{t}^{2} & =\beta_{0}+\beta_{1}\sigma_{t-1}^{2}\eta_{t-1}^{2}+\beta_{2}\sigma_{t-1}^{2} \end{array}$ where $\eta_{t}$ denotes standardized residuals and is assumed to be normally distributed. The values in parentheses denote the standard errors of the skewness and kurtosis, which can be calculated as $SE(skewness)=\sqrt{6/n}$ and $SE(excess\;kurtosis)=\sqrt{24/n}$, respectively. *** denote and 1% significance levels.

**Table 3:** Maximum likelihood estimates with various distributions for WTI and Brent crude oil returns.

Distribution	μ	σ	γ	p	q	L	LR
Panel A: WTI crude oil returns							
SGT	0.0169	2.6900 ***	−0.0466 ***	2.0458	1.5200 ***	−18,887.55	-
	0.03/(0.63)	0.08/(33.16)	0.01/(−3.19)	0.11/(0.42)	0.16/(9.33)		
ST	0.0172	2.6743 ***	−0.0465 ***	2	1.5867 ***	−18,887.64	0.18
	0.03/(0.64)	0.07/(39.23)	0.01/(−3.19)		0.06/(26.68)		
GT	0.0695 ***	2.5752 ***	0	1.8088 *	2.0100 ***	−18,895.89	16.68 ***
	0.02/(3.23)	0.06/(41.54)		0.12/(−1.65)	0.30/(6.71)		
T	0.0684 ***	2.6738 ***	0	2	1.5847 ***	−18,892.74	10.37 ***
	0.02/(3.18)	0.07/(39.22)			0.06/(26.69)		
Normal	0.0175	2.7970 ***	0	2	∞	−20,654.29	3533.48 ***
	0.03/(0.58)	0.02/(129.91)					
Panel B: Brent crude oil returns							
SGT	0.0178	2.4096 ***	−0.0327 **	1.8482	1.9809 ***	−18,492.56	-
	0.02/(0.71)	0.05/(46.35)	0.01/(−2.34)	0.09/(−1.69)	0.23/(8.60)		
ST	0.0166	2.4431 ***	−0.0340 **	2	1.6857 ***	−18,493.71	2.31
	0.02/(0.67)	0.05/(44.47)	0.01/(−2.39)		0.07/(25.20)		
GT	0.0517 **	2.4056 ***	0	1.8366 *	2.0100 ***	−18,495.30	5.49 **
	0.02/(2.55)	0.05/(46.99)		0.09/(−1.73)	0.24/(19.45)		
T	0.0506 **	2.4417 ***	0	2	1.6867 ***	−18,496.57	8.02 **
	0.02/(2.48)	0.05/(44.59)			0.07/(25.17)		
Normal	0.0174	2.5596 ***	0	2	∞	−20,049.55	3113.98 ***
	0.03/(0.63)	0.02/(130.38)					

Notes. This table shows the estimates of the SGT, ST, GT, T, and normal distributions for WTI and Brent crude oil returns. The sample period ranges from 4 January 1988 to 30 June 2021. The values in the second row of each distribution present the standard errors and t statistics in parentheses. L denotes the maximized log-likelihood value. LR gives the likelihood ratio statistic for the null hypothesis of the alternative distribution against the SGT distribution. *, **, and *** denote the 10%, 5%, and 1% significance levels, respectively.

**Table 4:** The results of ST-GARCH-type models with constant high-order moments for WTI and Brent crude oil returns.

Θ	GARCH	EGARCH	NAGARCH	AGARCH	GJR-GARCH	IGARCH	TGARCH	TS-GARCH
Panel A: WTI crude oil returns								
$\alpha_{0}$	0.0299	0.0134	0.0143	0.0157	0.0214	0.0268	0.0071	0.0357 *
	(1.45)	(0.60)	(0.68)	(0.75)	(1.03)	(1.29)	(0.34)	(1.81)
$\alpha_{1}$	−0.0251 **	−0.0248 **	−0.0227 **	−0.0231 **	−0.0244 **	−0.0253 **	−0.0238 **	−0.0277 ***
	(−2.29)	(−2.16)	(−2.07)	(−2.11)	(−2.22)	(−2.31)	(−2.34)	(−2.54)
$\beta_{0}$	0.0862 ***	−0.0952 ***	0.0841 ***	0.0745 ***	0.0861 ***	0.0606 ***	0.0309 ***	0.0335 ***
	(6.07)	(−11.85)	(6.17)	(4.99)	(6.14)	(5.97)	(5.69)	(5.61)
$\beta_{1}$	0.0834 ***	0.1558 ***	0.0784 ***	0.0810 ***	0.0654 ***		0.0605 ***	0.0901 ***
	(12.11)	(13.33)	(11.77)	(11.88)	(7.95)		(8.36)	(13.75)
$\beta_{2}$	0.9039 ***	0.9860 ***	0.9029 ***	0.9056 ***	0.9053 ***	0.9061 ***	0.9251 ***	0.9180 ***
	(124.66)	(432.52)	(125.80)	(124.91)	(126.64)	(130.27)	(155.40)	(145.56)
λ		−0.1900 ***	−0.2900 ***	−0.4299 ***	0.0320 ***		−0.0435 ***	
		(−4.04)	(−4.79)	(−3.83)	(3.00)		(−5.72)	
γ	−0.0747 ***	−0.0834 ***	−0.0792 ***	−0.0775 ***	−0.0773 ***	−0.0766 ***	−0.0854 ***	−0.0716 ***
	(−4.97)	(−5.45)	(−5.26)	(−5.16)	(−5.14)	(−5.01)	(−5.64)	(−4.76)
q	2.9521 ***	2.9681 ***	2.9828 ***	2.9665 ***	2.9562 ***	2.7302 ***	2.9456 ***	2.8774 ***
	(16.48)	(16.54)	(16.32)	(16.42)	(16.49)	(17.59)	(16.67)	(17.15)
L	−18,260.05	**−18,226.98**	−18,246.57	−18,252.20	−18,255.46	−18,265.86	−18,230.04	−18,245.84
AIC	36,536.09	**36,471.96**	36,511.14	36,522.39	36,528.92	36,545.72	36,478.08	36,507.69
BIC	36,592.42	**36,535.32**	36,574.50	36,585.76	36,592.29	36,595.01	36,541.45	36,564.01
Panel B: Brent crude oil returns								
$\alpha_{0}$	0.0301	0.0134	0.0176	0.0200	0.0224	0.0281	0.0095	0.0280
	(1.57)	(0.66)	(0.90)	(1.02)	(1.14)	(1.44)	(0.52)	(1.49)
$\alpha_{1}$	0.0287 ***	0.0291 ***	0.0306 ***	0.0265 **	0.0298 ***	0.0287 ***	0.0315 ***	0.0289 ***
	(2.65)	(2.67)	(2.82)	(2.43)	(2.74)	(2.65)	(2.86)	(2.75)
$\beta_{0}$	0.0574 ***	−0.0905 ***	0.0593 ***	0.0530 ***	0.0595 ***	0.0424 ***	0.0230 ***	0.0245 ***
	(5.16)	(−11.60)	(5.23)	(4.92)	(5.20)	(5.38)	(5.21)	(5.02)
$\beta_{1}$	0.0788 ***	0.1400 ***	0.0763 ***	0.0773 ***	0.0610 ***		0.0574 ***	0.0820 ***
	(11.78)	(12.69)	(11.52)	(12.06)	(8.25)		(9.35)	(12.98)
$\beta_{2}$	0.9131 ***	0.9900 ***	0.9094 ***	0.9129 ***	0.9124 ***	0.9148 ***	0.9312 ***	0.9274 ***
	(131.91)	(139.47)	(126.14)	(133.60)	(129.86)	(136.61)	(169.54)	(157.75)
λ		−0.1900 ***	−0.2752 ***	−0.1854 **	0.0351 ***		−0.0366 ***	
		(−4.17)	(−4.87)	(−1.97)	(3.53)		(−5.69)	
γ	−0.0473 ***	−0.0543 ***	−0.0497 ***	−0.0533 ***	−0.0480 ***	−0.0484 ***	−0.0537 ***	−0.0458 ***
	(−3.20)	(−3.66)	(−3.39)	(−3.66)	(−3.27)	(−3.25)	(−3.73)	(−3.11)
q	2.9917 ***	3.0192 ***	3.0849 ***	3.5340 ***	3.0581 ***	2.8326 ***	4.5665 ***	2.9645 ***
	(15.73)	(15.89)	(15.36)	(12.05)	(15.46)	(16.73)	(5.84)	(16.02)
L	−17,929.22	**−17,899.86**	−17,916.60	−17,926.90	−17,922.70	−17,932.19	−17,923.70	−17,920.42
AIC	35,874.44	**35,817.72**	35,851.20	35,871.81	35,863.37	35,878.38	35,865.40	35,856.84
BIC	35,930.82	**35,881.15**	35,914.63	35,935.24	35,926.80	35,927.72	35,928.83	35,913.23

Notes. The daily returns on the WTI and Brent crude oil prices range from 4 January 1988 to 30 June 2021. The mean equation is modeled as: $r_{t}=\alpha_{0}+\alpha_{1}r_{t-1}+\varepsilon_{t}$. The volatility equation is estimated with eight variations of the GARCH-type models. λ reflects asymmetry in volatility. The high-order moment parameters γ and q are constant. The values in parentheses and the last three rows present the t statistics, the maximized likelihood values, Akaike Information Criterion (AIC), and Bayesian Information Criterion (BIC), respectively. Bold values denote the best-performing models. *, **, and *** denote the 10%, 5%, and 1% significance levels, respectively.

**Table 5:** The results of ST-GARCH-type models with time-varying high-order moments for WTI crude oil returns.

Θ	GARCH	EGARCH	NAGARCH	AGARCH	GJR-GARCH	IGARCH	TGARCH	TS-GARCH
$\alpha_{0}$	0.0018	0.3938	0.4067 ***	0.4063 ***	0.4070 ***	0.0040 **	0.4079 ***	0.0090
	(0.04)	(0.30)	(16.95)	(19.43)	(22.44)	(2.19)	(15.54)	(0.63)
$\alpha_{1}$	−0.0115	−0.0007	−0.0330	−0.0162	−0.0110 ***	−0.0096 *	−0.0010 ***	−0.0232 ***
	(−1.04)	(−0.01)	(−0.86)	(−0.47)	(−5.55)	(−1.86)	(−3.20)	(−3.50)
$\beta_{0}$	0.1000 ***	0.5000 ***	0.0996 ***	0.0998 ***	0.0994 ***	0.1000 ***	0.0998 ***	0.1000 ***
	(14.00)	(3.02)	(17.35)	(21.38)	(3.78)	(23.26)	(4.11)	(22.30)
$\beta_{1}$	0.0899 ***	0.0877 ***	0.0899 ***	0.0897 ***	0.0898 ***		0.0890 ***	0.0898
	(37.02)	(12.80)	(65.63)	(45.76)	(8.62)		(4.36)	(30.06)
$\beta_{2}$	0.9100 ***	0.9896 ***	0.9099 ***	0.9099 ***	0.9100 ***	0.9100 ***	0.9100 ***	0.9100 ***
	(594.31)	(467.10)	(536.35)	(506.32)	(316.47)	(441.71)	(221.10)	(548.35)
λ		−0.0110 ***	−0.4976 ***	−0.4973 ***	0.4988 ***		−0.4988 ***	
		(−20.12)	(−85.65)	(−90.72)	(54.32)		(−54.00)	
$\gamma_{0}$	−0.0932 ***	−0.0001 **	−0.0064 ***	−0.0016 ***	−0.0034 ***	−0.0808 ***	−0.0004 ***	−0.1429 **
	(−3.12)	(−2.01)	(−2.48)	(−2.80)	(−2.84)	(−3.72)	(−2.67)	(−2.14)
$\gamma_{1}$	0.0269	0.1412 *	0.0058 ***	0.0230	0.0062 ***	0.0139 ***	0.0118 *	0.0190 ***
	(1.00)	(1.77)	(2.10)	(1.48)	(7.76)	(2.64)	(1.76)	(3.55)
$\gamma_{2}$	0.3940 **	0.4996 ***	0.3280 ***	0.2172 ***	0.2873 ***	0.4833 ***	0.2039 ***	0.2096 **
	(2.01)	(2.81)	(8.07)	(2.85)	(3.85)	(2.60)	(3.32)	(2.17)
$q_{0}$	−1.0255 *	−9.6515	−8.0437 ***	−8.7813	−9.4878 ***	−1.0611 ***	−7.7221 **	−9.2289 ***
	(−1.80)	(−1.59)	(−2.46)	(−1.42)	(−3.21)	(−2.58)	(−2.06)	(−4.80)
$q_{1}$	−0.2968 **	−0.0613 **	−0.0010 **	−0.1476 **	0.2551 ***	−0.2939 **	0.1544 **	0.0333
	(−2.14)	(−2.53)	(−2.13)	(−2.32)	(−3.09)	(−1.99)	(−3.08)	(−1.41)
$q_{2}$	0.1812	0.2806 ***	−0.0560 **	0.2329 **	0.0658 *	0.2107 ***	0.2795 ***	0.2486 ***
	(0.38)	(3.47)	(−2.02)	(2.38)	(1.89)	(4.47)	(5.59)	(6.12)
L	−11,024.70	**−5358.00**	−9418.65	−10,638.85	−7426.43	−11,021.6	−6097.76	−9903.59
AIC	22,073.40	**10,742.00**	18,863.30	21,303.70	14,878.87	22,065.10	12,221.52	19,831.18
BIC	22,157.89	**10,833.52**	18,954.83	21,395.23	14,970.40	22,142.55	12,313.05	19,915.67

Notes. The daily returns on the WTI crude oil prices range from 4 January 1988 to 30 June 2021. The conditional mean is modeled as: $r_{t}=\alpha_{0}+\alpha_{1}r_{t-1}+\varepsilon_{t}$. Conditional volatility is estimated with eight variations of the GARCH models. λ reflects asymmetry in volatility. The dynamics of high-order moment parameters $\tilde{\gamma }$ and $\tilde{q}$ are also assumed to follow a GARCH process. The values in parentheses and the last three rows present the t statistics, the maximized likelihood values, Akaike Information Criterion (AIC), and Bayesian Information Criterion (BIC), respectively. Bold values denote the best-performing models. *, **, and *** denote the 10%, 5%, and 1% significance levels, respectively.

**Table 6:** The results of ST-GARCH-type models with time-varying high-order moments for Brent crude oil returns.

Θ	GARCH	EGARCH	NAGARCH	AGARCH	GJR-GARCH	IGARCH	TGARCH	TS-GARCH
$\alpha_{0}$	0.0629	0.1310	0.4051 ***	0.4061 ***	0.4060 ***	0.0036 **	0.4069 ***	0.0338
	(1.16)	(0.21)	(24.18)	(18.13)	(24.20)	(0.06)	(17.08)	(2.11)
$\alpha_{1}$	0.1150 ***	0.1996 ***	0.1516 ***	0.0598	0.1782 ***	0.0722 *	0.1943 ***	0.1032 ***
	(3.40)	(3.48)	(3.91)	(1.56)	(9.51)	(1.58)	(4.49)	(4.53)
$\beta_{0}$	0.0999 ***	0.4989 ***	0.0999 ***	0.0999 ***	0.0999 ***	0.1000 ***	0.0983 ***	0.0995 ***
	(14.47)	(12.72)	(18.47)	(22.88)	(4.28)	(24.34)	(4.57)	(20.77)
$\beta_{1}$	0.0800 ***	0.0895 ***	0.0899 ***	0.0898 ***	0.0899 ***		0.0898 ***	0.0897
	(32.33)	(23.45)	(70.16)	(44.92)	(9.04)		(4.12)	(25.24)
$\beta_{2}$	0.9200 ***	0.9897 ***	0.9100 ***	0.9100 ***	0.9099 ***	0.9100 ***	0.9099 ***	0.9093 ***
	(674.86)	(377.73)	(516.87)	(504.98)	(313.00)	(455.21)	(210.51)	(550.20)
λ		−0.3727 ***	−0.4995 ***	−0.4970 ***	0.4996 ***		−0.4995 ***	
		(−33.16)	(−87.05)	(−90.72)	(34.37)		(−30.50)	
$\gamma_{0}$	−0.0343 ***	−0.0003 **	−0.0006 ***	−0.0031 ***	−0.0020 ***	−0.0606 ***	−0.0012 ***	−0.1770 **
	(−2.78)	(−2.04)	(−2.05)	(−2.47)	(−2.72)	(−2.32)	(−2.17)	(−3.22)
$\gamma_{1}$	0.1149	0.0046 *	0.0894 ***	0.025	0.2908 ***	0.0893 ***	0.1749 *	0.1324 ***
	(2.40)	(2.75)	(2.35)	(2.42)	(11.62)	(2.35)	(2.17)	(2.77)
$\gamma_{2}$	0.2430 ***	0.2469 ***	0.2073 ***	0.2912 ***	0.2038 ***	0.2443 ***	0.2017 ***	0.2462 **
	(1.54)	(2.68)	(5.06)	(4.50)	(2.97)	(2.35)	(2.09)	(2.36)
$q_{0}$	−2.8358 *	−9.5296	−7.0045 ***	−8.3612	−8.7497 ***	−1.4441 ***	−9.9497 **	−3.4405 ***
	(−2.07)	(−2.07)	(−2.81)	(−3.10)	(−2.11)	(−1.89)	(−2.09)	(−5.67)
$q_{1}$	−0.2719 **	0.0865 **	0.1424 **	−0.0540 **	0.2887 ***	−0.2877 **	0.1479 **	−0.1988
	(−2.88)	(1.73)	(2.05)	(−2.00)	(3.10)	(−1.33)	(3.00)	(−0.58)
$q_{2}$	−0.2786 ***	0.2812 ***	−0.0756 ***	0.0618 **	0.2464 *	0.1987 ***	0.1980 ***	−0.1448 ***
	(−3.37)	(3.03)	(−5.05)	(3.01)	(2.39)	(1.54)	(2.02)	(−0.41)
L	−10,865.02	−5397.06	−9387.15	−10,614.52	−7445.85	−11,014.44	−6099.39	−9939.07
AIC	21,754.05	**10,820.11**	18,800.30	21,255.03	14,917.69	22,050.88	12,224.77	19,902.15
BIC	21,838.62	**10,911.73**	18,891.93	21,346.66	15,009.31	22,128.40	12,316.39	19,986.72

Notes. The daily returns on Brent crude oil prices range from 4 January 1988 to 30 June 2021. The conditional mean is modeled as: $r_{t}=\alpha_{0}+\alpha_{1}r_{t-1}+\varepsilon_{t}$. Conditional volatility is estimated with eight variations of the GARCH models. λ reflects asymmetry in volatility. The dynamics of high-order moment parameters $\tilde{\gamma }$ and $\tilde{q}$ are also assumed to follow a GARCH process. The values in parentheses and the last three rows present the t statistics, the maximized likelihood values, Akaike Information Criterion (AIC), and Bayesian Information Criterion (BIC), respectively. Bold values denote the best-performing models. *, **, and *** denote the 10%, 5%, and 1% significance levels, respectively.

**Table 7:** In-sample conditional VaR performance of ST-GARCH-type models with time-varying high-order moments for WTI crude oil returns.

Θ	GARCH	EGARCH	NAGARCH	AGARCH	GJR-GARCH	IGARCH	TGARCH	TS-GARCH
0.5%	−3.3752	−3.2558	−3.3621	−3.2647	−3.2634	−3.221	−3.2878	−3.3958
N (0.5%)	34(42)	34(42)	33(42)	42(42)	34(42)	42(42)	40(42)	35(42)
LR^uc^/LR^cc^	1.71/4.01	1.71/4.01	2.18/4.58	0.00/1.58	1.71/4.01	0.00/1.58	0.12/0.50	1.31/3.50
1%	−2.7579	−2.6497	−2.7302	−2.6777	−2.6725	−2.6592	−2.6735	−2.76
N (1%)	66(84)	64(84)	66(84)	80(84)	75(84)	79(84)	86(84)	91(84)
LR^uc^/LR^cc^	4.38 **/6.89 **	5.43 **/8.13 **	4.38 **/6.89 **	0.23/1.67	1.10/1.24	0.36/0.44	0.03/1.11	0.51/1.34
2.5%	−2.0484	−1.9633	−2.014	−1.9675	−1.9779	−2.0036	−1.978	−2.0368
N (2.5%)	175(210)	194(210)	207(210)	200(210)	200(210)	211(210)	188(210)	223(210)
LR^uc^/LR^cc^	6.67 ***/8.03 **	1.44/3.84	0.08/0.24	2.43/3.12	2.43/3.12	0.01/0.10	2.66/5.55 *	0.69/5.89 *
5%	−1.5702	−1.5075	−1.5114	−1.5100	−1.5164	−1.5545	−1.5162	−1.5548
N (5%)	393(421)	395(421)	398(421)	414(421)	412(421)	384(421)	396(421)	454(421)
LR^uc^/LR^cc^	2.13/10.30 ***	1.85/9.73 ***	1.11/1.17	0.16/0.86	0.25/0.32	3.70 */6.87 **	1.71/1.83	2.50/2.51
10%	−1.1213	−1.0842	−1.0857	−1.0852	−1.088	−1.1265	−1.0876	−1.1065
N (10%)	840(843)	844(843)	846(843)	858(843)	837(843)	838(843)	850(843)	862(843)
LR^uc^/LR^cc^	0.02/1.03	0.00/0.82	0.01/0.96	0.26/0.51	0.06/0.77	0.05/0.38	0.05/0.13	0.43/1.37

Notes. This table shows statistics on the in-sample performance of eight variations of the ST-GARCH-type models. The skewness and kurtosis parameters are defined as a function of the innovation at period t−1 and an autoregressive process of order 1. The results are based on the daily returns of the WTI crude oil prices from 4 January 1988 to 30 June 2021 (8439 observations). The values in the first row in each panel are the average estimated thresholds. The values in the second row present the actual counts and the expected counts in parentheses (i.e., the number of observations falling in the 0.5%, 1%, 2.5%, 5%, and 10% tails of the return distribution). The last row gives the likelihood ratio (LR) statistics of the unconditional coverage test and conditional coverage test. *, **, and *** denote significance at the 10%, 5%, and 1% levels, respectively.

**Table 8:** In-sample conditional VaR performance of ST-GARCH-type models with time-varying high-order moments for Brent crude oil returns.

Θ	GARCH	EGARCH	NAGARCH	AGARCH	GJR-GARCH	IGARCH	TGARCH	TS-GARCH
0.5%	−3.2833	−3.2558	−3.2774	−3.2696	−3.2999	−3.3066	−3.2737	−3.2411
N (0.5%)	35(42)	36(42)	32(42)	42(42)	39(42)	49(42)	34(42)	34(42)
LR^uc^/LR^cc^	1.42/3.62	1.05/3.16	2.85 */5.37 *	0.00/1.58	0.30/2.13	0.95/2.07	1.83/4.14	1.83/4.14
1%	−2.6858	−2.6496	−2.6657	−2.6598	−2.6819	−2.7105	−2.6628	−2.6671
N (1%)	51(85)	69(85)	70(85)	81(85)	71(85)	71(85)	88(85)	67(85)
LR^uc^/LR^cc^	16.03 ***/17.03 ***	3.25 */3.54	2.84 */3.10	0.19/0.26	2.47/2.70	2.47/2.70	0.11/1.10	4.15 **/4.49
2.5%	−2.0006	−1.9633	−1.9732	−1.9695	−1.9825	−2.0228	−1.9713	−2.0014
N (2.5%)	141(212)	188(212)	185(212)	200(212)	213(212)	195(212)	188(212)	218(212)
LR^uc^/LR^cc^	27.94 ***/28.12 ***	3.01 */3.18	3.81 */3.81	0.00/0.02	0.77/0.79	1.52/1.58	3.01 */3.18	0.14/1.11
5%	−1.5397	−1.5075	−1.5133	−1.5111	−1.5183	−1.5576	−1.5121	−1.592
N (5%)	390(425)	420(425)	422(425)	421(425)	408(425)	380(425)	408(425)	471(425)
LR^uc^/LR^cc^	3.11 */3.11	0.06/0.23	0.02/0.50	0.04/1.35	0.73/1.49	5.19 **/5.19 *	0.73/0.87	5.07 **/5.11 *
10%	−1.1071	−1.0842	−1.0864	−1.0855	−1.0878	−1.119	−1.0858	−1.1196
N (10%)	845(850)	880(850)	861(850)	837(850)	876(850)	822(850)	876(850)	865(850)
LR^uc^/LR^cc^	0.03/0.39	1.16/1.49	0.15/1.14	0.22/1.55	0.88/1.13	1.04/2.12	0.88/0.98	0.29/0.78

Notes. This table shows statistics on the in-sample performance of eight variations of the ST-GARCH-type models. The skewness and kurtosis parameters are defined as a function of the innovation at period t−1 and autoregressive of order 1. The results are based on the daily returns of the Brent crude oil prices from 4 January 1988 to 30 June 2021 (8500 observations). The values in the first row in each panel are the average estimated thresholds. The values in the second row present the actual counts and the expected counts in parentheses (i.e., the number of observations falling in the 0.5%, 1%, 2.5%, 5%, and 10% tails of the return distribution). The last row gives the likelihood ratio (LR) statistics of the unconditional coverage test and conditional coverage test. *, **, and *** denote significance at the 10%, 5%, and 1% levels, respectively.

**Table 9:** Out-of-sample conditional VaR performance of ST-GARCH-type models with time-varying high-order moments for WTI crude oil returns.

Θ	GARCH	EGARCH	NAGARCH	AGARCH	GJR-GARCH	IGARCH	TGARCH	TS-GARCH
0.5%	−3.3084	−3.2564	−3.2706	−3.2282	−3.2638	−3.305	−3.2729	−3.4047
N (0.5%)	31(29)	28(29)	27(29)	38(29)	26(29)	33(29)	30(29)	31(29)
LR^uc^/LR^cc^	0.08/2.08	0.08/2.43	0.22/2.70	2.27/17.87 ***	0.43/3.05	0.41/2.19	0.01/2.12	0.08/6.66 **
1%	−2.7275	−2.6501	−2.6606	−2.6493	−2.6555	−2.7356	−2.6627	−2.7146
N (1%)	59(58)	52(58)	54(58)	67(58)	52(58)	59(58)	59(58)	56(58)
LR^uc^/LR^cc^	0.00/1.80	0.86/16.50 ***	0.43/3.45	1.06/3.86	0.86/4.23	0.00/1.80	0.00/1.80	0.15/1.94
2.5%	−2.0489	−1.9636	−1.97	−1.9828	−1.9824	−2.0643	−1.9655	−2.0288
N (2.5%)	155(147)	157(147)	146(147)	149(147)	133(147)	163(147)	157(147)	149(147)
LR^uc^/LR^cc^	0.40/22.27 ***	0.63/2.83	0.01/2.51	0.01/2.50	1.49/3.77	1.64/2.05	0.63/2.83	0.02/3.99
5%	−1.5836	−1.5128	−1.5114	−1.5324	−1.5096	−1.5997	−1.5127	−1.5451
N (5%)	284(294)	282(294)	294(294)	288(294)	282(294)	292(294)	285(294)	282(294)
LR^uc^/LR^cc^	0.42/3.31	0.59/3.02	0.00/2.98	0.17/3.46	0.59/3.02	0.02/3.29	0.35/2.89	0.59/3.02
10%	−1.1399	−1.0861	−1.0856	−1.1077	−1.0887	−1.1492	−1.0846	−1.1033
N (10%)	567(589)	598(589)	609(589)	596(589)	581(589)	570(589)	598(589)	575(589)
LR^uc^/LR^cc^	0.97/3.43	0.13/2.90	0.70/2.97	0.08/2.08	0.14/2.29	0.73/2.18	0.13/2.77	0.40/2.50

Notes. This table shows statistics on the out-of-sample performance of eight variations of the GARCH model with the ST distribution. The skewness and kurtosis parameters are defined as a function of the innovation at period t−1 and autoregressive of order 1. The results are based on the daily returns of the WTI crude oil price from 4 January 1988 to 30 June 2021 (8439 observations). A 10-year rolling sample, starting from 4 January 1988, is used to estimate conditional VaR measures, and a 1-year holdout sample is used to evaluate their performance. The values in the first row in each panel are the average estimated thresholds. The values in the second row present the actual counts and the expected counts in parentheses (i.e., the number of observations falling in the 0.5%, 1%, 2.5%, 5%, and 10% tails of the return distribution). The last row gives the likelihood ratio (LR) statistics of the unconditional coverage test and conditional coverage test. **, and *** denote significance at the 5%, and 1% levels, respectively.

**Table 10:** Out-of-sample conditional VaR performance of ST-GARCH-type models with time-varying high-order moments for Brent crude oil returns.

Θ	GARCH	EGARCH	NAGARCH	AGARCH	GJR-GARCH	IGARCH	TGARCH	TS-GARCH
0.5%	−3.2564	−3.2734	−3.2715	−3.2093	−3.2608	−3.2369	−3.2597	−3.3327
N (0.5%)	36(29)	27(29)	27(29)	29(29)	27(29)	26(29)	25(29)	29(29)
LR^uc^/LR^cc^	1.21/3.52	0.27/2.95	0.27/2.95	0.02/2.77	0.27/2.95	0.51/3.06	0.83/2.34	0.02/2.77
1%	−2.6884	−2.6627	−2.6613	−2.6403	−2.6533	−2.6837	−2.6525	−2.7059
N (1%)	61(59)	63(59)	55(59)	54(59)	63(59)	52(59)	46(59)	56(59)
LR^uc^/LR^cc^	0.03/22.52 ***	0.19/2.18	0.37/2.02	0.55/2.08	0.19/2.18	1.02/1.68	3.41/2.16	0.23/3.96
2.5%	−2.0247	−1.9713	−1.9704	−1.9823	−1.9656	−2.0315	−1.9651	−1.9969
N (2.5%)	141(149)	159(149)	122(149)	159(149)	151(149)	149(149)	159(149)	122(149)
LR^uc^/LR^cc^	0.45/17.90 ***	0.67/3.30	5.36 **/9.17 **	0.67/3.30	0.03/1.47	0.00/1.52	0.67/2.33	5.36 **/6.92 **
5%	−1.5692	−1.5121	−1.5116	−1.5355	−1.5088	−1.5085	−1.5127	−1.5265
N (5%)	313(298)	286(298)	307(298)	292(298)	286(298)	253(298)	308(298)	290(298)
LR^uc^/LR^cc^	0.77/2.77	0.52/1.19	0.28/8.96 **	0.13/5.25 *	0.52/3.87	7.55 ***/21.15 ***	0.35/2.89	0.23/2.29
10%	−1.1343	−1.0859	−1.0857	−1.1124	−1.0846	−1.1452	−1.0845	−1.0906
N (10%)	601(596)	602(596)	602(596)	622(596)	599(596)	599(596)	579(596)	604(596)
LR^uc^/LR^cc^	0.04/1.53	0.06/1.46	0.06/1.46	1.22/15.94 **	0.01/1.43	0.01/1.43	0.56/2.66	0.11/2.34

Notes. This table shows statistics on the out-of-sample performance of eight variations of the GARCH model with the ST distribution. The skewness and kurtosis parameters are defined as a function of the innovation at period t−1 and autoregressive of order 1. The results are based on the daily returns of the Brent crude oil price from 4 January 1988 to 30 June 2021 (8500 observations). A 10-year rolling sample, starting from 4 January 1988, is used to estimate conditional VaR measures, and a 1-year holdout sample is used to evaluate their performance. The values in the first row in each panel are the average estimated thresholds. The values in the second row present the actual counts and the expected counts in parentheses (i.e., the number of observations falling in the 0.5%, 1%, 2.5%, 5%, and 10% tails of the return distribution). The last row gives the likelihood ratio (LR) statistics of the unconditional coverage test and conditional coverage test. *, **, and *** denote significance at the 10%, 5%, and 1% levels, respectively.

**Table 11:** Out-of-sample conditional VaR performance with the historical simulation method for WTI and Brent crude oil returns.

Θ	WTI	Brent
0.5%	−2.7092	−2.7455
N (0.5%)	47(29)	47(29)
LR^uc^/LR^cc^	8.86 ***/59.59 ***	8.47 ***/32.05 ***
1%	−2.4876	−2.4898
N (1%)	75(58)	85(59)
LR^uc^/LR^cc^	4.06 **/40.63 ***	9.64 ***/36.20 ***
2.5%	−1.8779	−1.7381
N (2.5%)	181(147)	187(149)
LR^uc^/LR^cc^	7.33 ***/35.13 ***	9.18 ***/26.96 ***
5%	−1.7512	−1.5556
N (5%)	339(294)	348(298)
LR^uc^/LR^cc^	6.67 ***/37.30 ***	8.36 ***/32.62 ***
10%	−0.7657	−0.7845
N (10%)	640(589)	643(596)
LR^uc^/LR^cc^	4.67 **/32.19 ***	3.99 **/22.43 ***

Notes. This table shows statistics on the out-of-sample performance with the historical simulation method for WTI and Brent crude oil returns. A 10-year rolling sample, starting from 4 January 1988, is used to estimate conditional VaR measures, and a 1-year holdout sample is used to evaluate their performance. The values in the first row in each panel are the average estimated thresholds. The values in the second row present the actual counts and the expected counts in parentheses (i.e., the number of observations falling in the 0.5%, 1%, 2.5%, 5%, and 10% tails of the return distribution). The last row gives the likelihood ratio (LR) statistics of the unconditional coverage test and conditional coverage test. **, and *** denote significance at the 5%, and 1% levels, respectively.

**Table 12:** Out-of-sample conditional VaR performance of ST-GARCH-type models with time-varying skewness or kurtosis parameters for WTI and Brent crude oil returns.

Θ	WTI				Brent			
	GARCH-CS	GARCH-CK	EGARCH-CS	EGARCH-CK	GARCH-CS	GARCH-CK	EGARCH-CS	EGARCH-CK
0.5%	−3.3318	−3.3841	−3.3471	−3.2638	−3.2827	−3.2647	−3.2664	−3.2669
N (0.5%)	31(29)	9(29)	28(29)	7(29)	38(29)	7(29)	37(29)	7(29)
LR^uc^/LR^cc^	0.08/2.08	19.67 ***/19.70 ***	0.08/0.31	24.92 ***/24.92 ***	2.08/4.28	25.42 ***/26.21 ***	1.62/3.87	25.42 ***/26.21 ***
1%	−2.7462	−2.7443	−2.7418	−2.6564	−2.7070	−2.6564	−2.6937	−2.6584
N (1%)	57(58)	15(58)	28(58)	10(58)	61(59)	11(59)	61(59)	11(59)
LR^uc^/LR^cc^	0.07/1.89	47.19 ***/47.26 ***	20.38 ***/20.62 ***	62.84 ***/62.85 ***	0.03/22.81 ***	60.46 ***/61.97 ***	0.03/22.81 ***	60.46 ***/61.97 ***
2.5%	−2.0616	−2.0206	−1.9655	−1.9683	−2.0351	−1.9676	−2.0259	−1.9692
N (2.5%)	140(147)	43(147)	107(147)	30(147)	138(149)	19(149)	140(149)	20(149)
LR^uc^/LR^cc^	0.58/2.33	104.74 ***/113.26 ***	14.83 ***/48.49 ***	141.66 ***/142.35 ***	0.86/26.91 ***	184.71 ***/186.56 ***	0.58/23.53 ***	180.60 ***/182.39 ***
5%	−1.5918	−1.5408	−1.5214	−1.5110	−1.5746	−1.5101	−1.5685	−1.5114
N (5%)	284(294)	82(294)	228(294)	62(294)	307(298)	49(298)	310(298)	50(298)
LR^uc^/LR^cc^	0.19/25.51 ***	223.73 ***/256.40 ***	14.28 ***/41.45 ***	281.81 ***/285.70 ***	0.28/2.08	332.05 ***/333.74 ***	0.49/2.58	328.37 ***/330.02 ***
10%	−1.1437	−1.0965	−1.1788	−1.0861	−1.1355	−1.0852	−1.1325	−1.0860
N (10%)	577(589)	196(589)	528(589)	154(589)	588(596)	125(596)	594(596)	126(596)
LR^uc^/LR^cc^	0.21/13.19 ***	383.98 ***/409.19 ***	20.00 ***/20.10 ***	633.95 ***/655.08 ***	0.12/1.93	618.62 ***/686.17 ***	0.01/1.66	613.53 ***/681.89 ***

Notes. This table reports the out-of-sample performance statistics of the TS-GARCH and TS-EGARCH models with time-varying skewness or kurtosis parameters for WTI and Brent crude oil returns, where CS denotes constant skewness with time-varying kurtosis, and CK denotes constant kurtosis with time-varying skewness. The skewness or kurtosis parameters are defined as a function of the innovation at period t−1 and autoregressive of order 1. The results are based on the daily returns of WTI and Brent crude oil prices from 4 January 1988 to 30 June 2021 (8439 and 8500 observations, respectively). A 10-year rolling sample, starting from 4 January 1988, is used to estimate the conditional VaR measures, and a 1-year holdout sample is used to evaluate their performance. The values in the first row of each panel are the average estimated thresholds. The values in the second row present the actual counts, with the expected counts in parentheses (i.e., the number of observations falling in the 0.5%, 1%, 2.5%, 5%, and 10% tails of the return distribution). The last row reports the likelihood ratio (LR) statistics of the unconditional coverage test and the conditional coverage test. and *** denote significance at the and 1% levels, respectively.

**Table 13:** ES backtests based on ST-GARCH and ST-EGARCH models with time-varying skewness and kurtosis parameters for WTI and Brent crude oil returns.

GARCH-Type Models	GARCH	EGARCH
Panel A: WTI crude oil returns		
0.5%	−4.4093(0.20)	−4.4767(0.92)
1%	−3.6942(0.18)	−3.6956(0.17)
5%	−2.3313(0.12)	−2.2765(0.34)
Panel B: Brent crude oil returns		
0.5%	−4.3331(0.27)	−4.4996(0.15)
1%	−3.6338(0.24)	−3.7127(0.16)
5%	−2.3008(0.19)	−2.2751(0.34)

Notes. This table reports ES thresholds and *p*-values of the ES test (in parentheses) for the out-of-sample performance of the GARCH and EGARCH models with the ST distribution. The skewness and kurtosis parameters are defined as a function of the innovation at period *t* − 1 and autoregressive of order 1.

## Data Availability

Daily spot prices for WTI and Brent crude oil were obtained from the U.S. Energy Information Administration (EIA, https://www.eia.gov/dnav/pet/pet_pri_spt_s1_d.htm), accessed on 15 July 2025. The code used for estimation is available from the corresponding author upon reasonable request.

## Appendix A. Descriptive Statistics of the Holdout Sample Sizes for WTI and Brent Crude Oil Returns

Table A1 summarizes the holdout sample sizes for both WTI and Brent crude oil returns. We observe that 24 years of holdout samples are utilized to evaluate the out-of-sample conditional VaR performance, and the statistics are almost the same for both WTI and Brent crude oil returns. It is important to note that, for the last year, only 6 months’ returns are evaluated as out-of-sample, given that the sample spans from 4 January 1988 to 30 June 2021. This results in the minimum holdout sample sizes being 124 for both WTI and Brent crude oil returns.

**Table A1:** Descriptive statistics of the holdout sample sizes for WTI and Brent crude oil returns.

Crude Oil Market	N	Mean	Median	Minimum	Maximum
WTI	24	246	251	124	253
Brent	24	248	254	124	261

Notes. This table shows the descriptive statistics of the holdout sample sizes for WTI and Brent crude oil returns. N denotes the number of years of holdout samples in total. The holdout sample period ranges from 2 January 1998 to 30 June 2021.

## Appendix B. Evaluation of Out-of-Sample Conditional VaR with Conventional GARCH-Type Models

Table A2 and Table A3 provide the unconditional and conditional coverage test statistics on the out-of-sample VaR performance of conventional GARCH-type models for both WTI and Brent crude oil returns. For WTI crude oil returns, eight unconditional coverage (LR^uc^) test statistics and one conditional coverage (LR^cc^) test statistic cannot reject the null hypothesis at least at the 5% level. For Brent crude oil returns, thirteen unconditional coverage (LR^uc^) test statistics cannot reject the null hypothesis at least at the 5% level. All these results indicate superior performance of the conditional VaR approach introduced in this paper as compared to the conventional GARCH-type models.

## Appendix C. Conditional Pearson’s Type IV and Johnson’s SU Distributions with Time-Varying Parameters

### Appendix C.1. Conditional Pearson’s Type IV Distribution with Time-Varying Parameters

The probability density function of Pearson’s Type IV distribution can be written as follows:

(A1) $$f_{pearsonIV}\left(r;\mu ,\sigma ,\;m,\;\upsilon \right)=\frac{{\left|\frac{\Gamma (m+\frac{\upsilon }{2}i)}{\Gamma (m)}\right|}^{2}}{\sigma B(m-\frac{1}{2},\frac{1}{2})}{\left[1+{\left(\frac{r-\mu }{\sigma }\right)}^{2}\right]}^{-m}e^{-\upsilon arctan\left(\frac{r-\mu }{\sigma }\right)}$$

where Γ(·) and B(·) are the Gamma function and Beta function, μ is the location parameter, σ>0 is the scale parameter, $m>\frac{1}{2}$ and υ are the shape parameters.

Similarly, we allow the shape parameters in Pearson’s Type IV distribution to be a function of historical information as follows:

(A2) $${\tilde{m}}_{t}=m_{0}+m_{1}\eta_{t-1}+m_{2}{\tilde{m}}_{t-1},$$

(A3) $${\tilde{\upsilon }}_{t}=\upsilon_{0}+\upsilon_{1}\eta_{t-1}+\upsilon_{2}{\tilde{\upsilon }}_{t-1},$$

where ${\tilde{m}}_{t}$ and ${\tilde{\upsilon }}_{t}$ are the unrestricted shape parameters. According to the definition of Pearson’s Type IV density, we impose the restriction of

(A4) $$m_{t}=1/2+exp({\tilde{m}}_{t})$$

Then, the conditional Pearson’s Type IV GARCH parameters are obtained from the maximization of the sample log-likelihood function as follows:

(A5) $$\begin{array}{c} L=\sum_{t=1}^{T}\ln\left(f\left(\eta_{t}|m_{t},{\tilde{\upsilon }}_{t}\right)\right) \end{array}$$

with respect to the parameters in the dynamic system.

### Appendix C.2. Conditional Johnson’s SU Distribution with Time-Varying Parameters

The probability density function of Johnson’s SU is

(A6) $$f_{JohnsonSU}\left(r;\mu ,\sigma ,\;\xi ,\;\tau \right)=\frac{\tau }{\sigma \sqrt{1+{\left(\frac{r-\mu }{\sigma }\right)}^{2}}}\phi [\xi +\tau sinh^{-1}\left(\frac{r-\mu }{\sigma }\right)],$$

where ϕ is the density function of the standard normal distribution N(0,1), μ and σ>0 are location and scale parameters, respectively, ξ can be a skewness parameter, and τ>0 can be interpreted as a kurtosis parameter.

Then, let shape parameters ξ and τ in Johnson’s SU distribution follow a function of historical information as follows:

(A7) $${\tilde{\xi }}_{t}=\xi_{0}+\xi_{1}\eta_{t-1}+\xi_{2}{\tilde{\xi }}_{t-1},$$

(A8) $${\tilde{\tau }}_{t}=\tau_{0}+\tau_{1}\eta_{t-1}+\tau_{2}{\tilde{\tau }}_{t-1},$$

where ${\tilde{\xi }}_{t}$ and ${\tilde{\tau }}_{t}$ are the unrestricted shape parameters. According to the definition of Johnson’s SU density, we impose the restriction of

(A9) $$\tau_{t}=exp({\tilde{\tau }}_{t})$$

Then, the conditional Johnson’s SU GARCH parameters are obtained from the maximization of the sample log-likelihood function as follows:

(A10) $$\begin{array}{c} L=\sum_{t=1}^{T}\ln\left(f\left(\eta_{t}|{\tilde{\xi }}_{t},\tau_{t}\right)\right) \end{array}$$

with respect to the parameters in the dynamic system.

## Appendix D. Evaluation of Out-of-Sample Conditional VaR Performance of GARCH-Type Models with Standardized Residuals Following Pearson’s Type IV and Johnson’s SU Distributions

Table A4, Table A5, Table A6 and Table A7 report the unconditional and conditional coverage test statistics for the out-of-sample VaR forecasting performance of GARCH-type models with standardized residuals following Pearson’s Type IV and Johnson’s SU distributions. Specifically, Table A4 and Table A5 demonstrate that for models with Pearson’s Type IV distribution, both the LR^uc^ and LR^cc^ tests reject the null hypothesis even at the 1% significance level for both WTI and Brent crude oil returns. In contrast, models under Johnson’s SU distribution (Table A6 and Table A7) exhibit improved performance at low coverage levels. Nevertheless, the ST-GARCH-type models proposed in this study consistently outperform their counterparts based on both candidate distributions.

**Table A2:** Out-of-sample conditional VaR performance of conventional GARCH-type models for WTI crude oil returns.

Θ	GARCH	EGARCH	NAGARCH	AGARCH	GJR-GARCH	IGARCH	TGARCH	TS-GARCH
0.5%	−3.3637	−2.9070	−3.0119	−3.0209	−2.8788	−3.3195	−3.1301	−3.571
N (0.5%)	46(29)	9(29)	49(29)	48(29)	10(29)	33(29)	41(29)	31(29)
LR^uc^/LR^cc^	7.94 ***/15.71 ***	19.67 ***/19.70 ***	10.82 ***/17.90 ***	9.82 ***/21.77 ***	17.40 ***/17.44 ***	0.41/6.51 **	4.03 **/13.09 ***	0.08/6.66 **
1%	−2.7638	−2.2927	−2.3729	−2.4798	−2.2726	−2.7464	−2.4508	−2.7998
N (1%)	68(58)	19(58)	87(58)	78(58)	21(58)	58(58)	77(58)	63(58)
LR^uc^/LR^cc^	1.33/36.48 ***	37.16 ***/40.96 ***	11.75 ***/41.88 ***	5.64 **/40.48 ***	32.81 ***/36.23 ***	0.02/1.85	5.09 **/40.49 ***	0.27/16.81 ***
2.5%	−2.0664	−1.6488	−1.7009	−1.8639	−1.6371	−2.0709	−1.7399	−1.9759
N (2.5%)	157(147)	55(147)	215(147)	183(147)	59(147)	120(147)	197(147)	157(147)
LR^uc^/LR^cc^	0.62/21.84 ***	77.84 ***/97.52 ***	27.92 ***/51.93 ***	8.20 ***/2.95 ***	70.11 ***/111.10 ***	5.57 **/29.90 ***	15.51 ***/43.19 ***	0.63/21.84 ***
5%	−1.5906	−1.2533	−1.287	−1.4518	−1.2466	−1.6035	−1.3047	−1.4614
N (5%)	288(294)	121(294)	452(294)	335(294)	132(294)	224(294)	419(294)	313(294)
LR^uc^/LR^cc^	0.17/27.65 ***	137.44 ***/165.16 ***	76.41 ***/96.23 ***	5.54 **/42.03 ***	118.16 ***/145.15 ***	19.45 ***/45.51 ***	49.00 ***/74.23 ***	1.16/32.87 ***
10%	−1.1390	−0.9084	−0.9255	−1.0653	−0.9058	−1.1545	−0.9271	−1.0091
N (10%)	655(589)	275(589)	795(589)	641(589)	284(589)	493(589)	796(589)	643(589)
LR^uc^/LR^cc^	7.81 ***/36.50 ***	228.02 ***/257.89 ***	77.50 ***/95.46 ***	4.86 **/30.81 ***	213.56 ***/244.79 ***	18.52 ***/40.35 ***	73.18 ***/93.91 ***	5.24 **/28.18 ***

Notes. This table shows statistics on the out-of-sample performance of eight variations of the conventional GARCH model. The results are based on the daily returns of the WTI crude oil price from 4 January 1988 to 30 June 2021 (8439 observations). A 10-year rolling sample, starting from 4 January 1988, is used to estimate conditional VaR measures, and a 1-year holdout sample is used to evaluate their performance. The values in the first row in each panel are the average estimated thresholds. The values in the second row present the actual counts and the expected counts in parentheses (i.e., the number of observations falling in the 0.5%, 1%, 2.5%, 5%, and 10% tails of the return distribution). The last row gives the likelihood ratio (LR) statistics of the unconditional coverage test and conditional coverage test. **, and *** denote significance at the 5%, and 1% levels, respectively.

**Table A3:** Out-of-sample conditional VaR performance of conventional GARCH-type models for Brent crude oil returns.

Θ	GARCH	EGARCH	NAGARCH	AGARCH	GJR-GARCH	IGARCH	TGARCH	TS-GARCH
0.5%	−3.3055	−2.8620	−2.9415	−2.9464	−2.8091	−3.2593	−3.1136	−3.5145
N (0.5%)	45(29)	11(29)	42(29)	39(29)	12(29)	25(29)	26(29)	22(29)
LR^uc^/LR^cc^	6.72 ***/25.31 ***	15.75 ***/30.91 ***	4.44 **/24.41 ***	2.59/24.07 ***	13.83 **/38.43 ***	0.83/23.46 ***	0.51/22.48 ***	2.26/18.90 ***
1%	−2.7135	−2.2607	−2.3243	−2.4229	−2.2231	−2.6967	−2.4391	−2.7533
N (1%)	76(59)	16(59)	78(59)	69(59)	20(59)	52(59)	57(59)	54(59)
LR^uc^/LR^cc^	4.18 **/20.81 ***	45.47 ***/66.21 ***	5.22 **/25.52 ***	1.42/20.58 ***	35.82 ***/53.66 ***	1.03/16.76 ***	0.12/24.49 ***	0.55/20.79 ***
2.5%	−2.0291	−1.6302	−1.6742	−1.8275	−1.6083	−2.0328	−1.7332	−1.9431
N (2.5%)	138(149)	47(149)	221(149)	164(149)	50(149)	95(149)	150(149)	139(149)
LR^uc^/LR^cc^	0.86/19.22 ***	97.39 ***/115.11 ***	31.09 ***/44.53 ***	1.49/13.30 ***	90.55 ***/112.63 ***	23.02 ***/41.38 ***	0.01/14.91 ***	0.71/18.76 ***
5%	−1.5644	−1.2428	−1.2728	−1.4289	−1.2302	−1.5769	−1.3009	−1.4389
N (5%)	309(298)	91(298)	477(298)	362(298)	111(298)	234(298)	391(298)	337(298)
LR^uc^/LR^cc^	0.41/23.57 ***	205.73 ***/225.48 ***	96.37 ***/110.43 ***	13.54 ***/33.57 ***	161.00 ***/181.44 ***	15.61 ***/31.98 ***	27.87 ***/43.30 ***	5.14 **/25.48 ***
10%	−1.1246	−0.9045	−0.9214	−1.0549	−0.8997	−1.1402	−0.9258	−0.9968
N (10%)	754(596)	282(596)	843(596)	677(596)	306(596)	519(596)	747(596)	675(596)
LR^uc^/LR^cc^	43.19 ***/56.78 ***	224.20 ***/236.40 ***	101.94 ***/111.52 ***	11.71 ***/30.20 ***	187.62 ***/209.73 ***	11.56 ***/29.45 ***	39.56 ***/54.82 ***	16.14 ***/26.29 ***

Notes. This table shows statistics on the out-of-sample performance of eight variations of the conventional GARCH model. The results are based on the daily returns of the Brent crude oil price from 4 January 1988 to 30 June 2021 (8500 observations). A 10-year rolling sample, starting from 4 January 1988, is used to estimate conditional VaR measures, and a 1-year holdout sample is used to evaluate their performance. The values in the first row in each panel are the average estimated thresholds. The values in the second row present the actual counts and the expected counts in parentheses (i.e., the number of observations falling in the 0.5%, 1%, 2.5%, 5%, and 10% tails of the return distribution). The last row gives the likelihood ratio (LR) statistics of the unconditional coverage test and conditional coverage test. **, and *** denote significance at the 5%, and 1% levels, respectively.

**Table A4:** Out-of-sample conditional VaR performance of GARCH-type models with standardized residuals following Pearson’s Type IV distribution for WTI crude oil returns.

Θ	GARCH	EGARCH	NAGARCH	AGARCH	GJR-GARCH	IGARCH	TGARCH	TS-GARCH
0.5%	−8.5802	−8.3140	−7.8986	−8.2808	−8.6569	−7.9291	−8.3780	−8.0796
N (0.5%)	6(29)	6(29)	6(29)	6(29)	6(29)	2(29)	6(29)	5(29)
LR^uc^/LR^cc^	27.95 ***/27.96 ***	27.95 ***/27.96 ***	27.95 ***/27.96 ***	27.95 ***/27.96 ***	27.95 ***/27.96 ***	44.33 ***/44.33 ***	27.95 ***/27.96 ***	31.32 ***/31.33 ***
1%	−5.9445	−5.7359	−5.4202	−5.9149	−6.0452	−6.4967	−5.3955	−5.6020
N (1%)	9(58)	8(58)	8(58)	9(58)	9(58)	6(58)	11(58)	7(58)
LR^uc^/LR^cc^	66.51 ***/66.54 ***	70.41 ***/70.43 ***	70.41 ***/70.43 ***	66.51 ***/83.26 ***	66.51 ***/66.54 ***	78.98 ***/78.99 ***	59.37/59.42	74.55 ***/74.56 ***
2.5%	−3.5557	−3.4746	−3.2586	−3.5218	−3.7130	−3.9736	−3.4808	−3.4114
N (2.5%)	39(147)	33(147)	46(147)	40(147)	5(147)	12(147)	35(147)	24(147)
LR^uc^/LR^cc^	115.12 ***/119.99 ***	132.28 ***/138.39 ***	97.44 ***/101.15 ***	112.45 ***/117.14 ***	254.45 ***/254.47 ***	213.77 ***/213.82 ***	126.34 ***/127.93 ***	162.31 ***/165.22 ***
5%	−2.3312	−2.3307	−2.1701	−2.2637	−2.5132	−3.1411	−3.6835	−2.2952
N (5%)	119(294)	106(294)	133(294)	126(294)	31(294)	35(294)	73(294)	74(294)
LR^uc^/LR^cc^	141.15 ***/169.72 ***	167.05 ***/181.80 ***	116.51 ***/150.64 ***	128.43 ***/157.91 ***	400.19 ***/412.53 ***	382.30 ***/387.96 ***	248.48 ***/261.74 ***	245.62 ***/277.37 ***
10%	−1.4915	−1.5030	−1.3708	−1.4430	−1.6273	−2.2931	−1.8281	−1.4761
N (10%)	327(589)	311(589)	379(589)	362(589)	107(589)	104(589)	207(589)	232(589)
LR^uc^/LR^cc^	152.46 ***/185.47 ***	173.71 ***/195.73 ***	94.48 ***/117.30 ***	111.66 ***/150.30 ***	342.33 ***/352.62 ***	360.13 ***/388.13 ***	358.80 ***/391.31 ***	306.00 ***/326.98 ***

Notes. This table shows statistics on the out-of-sample performance of eight variations of the GARCH model with Pearson’s Type IV distribution. The skewness and kurtosis parameters are defined as a function of the innovation at period t−1 and autoregressive of order 1. The results are based on the daily returns of the WTI crude oil price from 4 January 1988 to 30 June 2021 (8439 observations). A 10-year rolling sample, starting from 4 January 1988, is used to estimate conditional VaR measures, and a 1-year holdout sample is used to evaluate their performance. The values in the first row in each panel are the average estimated thresholds. The values in the second row present the actual counts and the expected counts in parentheses (i.e., the number of observations falling in the 0.5%, 1%, 2.5%, 5%, and 10% tails of the return distribution). The last row gives the likelihood ratio (LR) statistics of the unconditional coverage test and conditional coverage test. *** denote significance at the 1% levels.

**Table A5:** Out-of-sample conditional VaR performance of GARCH-type models with standardized residuals following Pearson’s Type IV distribution for Brent crude oil returns.

Θ	GARCH	EGARCH	NAGARCH	AGARCH	GJR-GARCH	IGARCH	TGARCH	TS-GARCH
0.5%	−8.1356	−7.6105	−8.2122	−8.0109	−7.2766	−9.0848	−8.0906	−7.8541
N (0.5%)	6(29)	5(29)	1(29)	6(29)	1(29)	1(29)	6(29)	4(29)
LR^uc^/LR^cc^	28.47 ***/28.49 ***	31.86 ***/31.88 ***	50.97 ***/50.97 ***	28.48 ***/28.49 ***	50.97 ***/50.97 ***	50.97 ***/50.97 ***	28.48 ***/28.49 ***	35.66 ***/35.67 ***
1%	−5.5600	−5.2458	−5.6141	−5.4702	−5.0813	−6.3430	−5.7840	−5.4577
N (1%)	11(59)	10(59)	3(59)	11(59)	5(59)	5(59)	3(59)	7(59)
LR^uc^/LR^cc^	60.46 ***/75.62 ***	63.95 ***/79.95 ***	95.85 ***/95.85 ***	60.46 ***/86.26 ***	84.96 ***/84.97 ***	84.96 ***/84.97 ***	95.85 ***/95.85 ***	75.72 ***/83.63 ***
2.5%	−3.3285	−3.1890	−3.3477	−3.2765	−3.1188	−3.8960	−3.2260	−3.3317
N (2.5%)	35(149)	30(149)	27(149)	39(149)	10(149)	10(149)	39(149)	19(149)
LR^uc^/LR^cc^	128.90 ***/145.92 ***	144.33 ***/163.89 ***	154.39 ***/156.89 ***	117.59 ***/132.87 ***	227.37 ***/243.37 ***	227.37 ***/243.37 ***	107.43 ***/123.33 ***	184.71 ***/203.22 ***
5%	−2.2121	−2.1462	−2.2236	−2.1803	−2.1046	−2.6398	−2.5718	−2.2445
N (5%)	117(298)	92(298)	112(298)	128(298)	40(298)	23(298)	26(298)	62(298)
LR^uc^/LR^cc^	149.08 ***/161.07 ***	203.29 ***/218.78 ***	158.97 ***/166.45 ***	128.84 ***/147.12 ***	367.10 ***/381.98 ***	445.50 ***/461.58 ***	405.50 ***/405.43 ***	287.19 ***/309.24 ***
10%	−1.3989	−1.4010	−1.4728	−1.3939	−1.3480	−1.7160	−1.6757	−1.4453
N (10%)	412(596)	338(596)	354(596)	421(596)	125(596)	91(596)	111(596)	223(596)
LR^uc^/LR^cc^	70.14 ***/84.60 ***	144.98 ***/157.11 ***	126.11 ***/143.44 ***	63.09 ***/82.19 ***	456.30 ***/473.23 ***	479.12 ***/493.85 ***	458.58 ***/483.57 ***	333.18 ***/344.24 ***

Notes. This table shows statistics on the out-of-sample performance of eight variations of the GARCH model with Pearson’s Type IV distribution. The skewness and kurtosis parameters are defined as a function of the innovation at period t−1 and autoregressive of order 1. The results are based on the daily returns of the Brent crude oil price from 4 January 1988 to 30 June 2021 (8500 observations). A 10-year rolling sample, starting from 4 January 1988, is used to estimate conditional VaR measures, and a 1-year holdout sample is used to evaluate their performance. The values in the first row in each panel are the average estimated thresholds. The values in the second row present the actual counts and the expected counts in parentheses (i.e., the number of observations falling in the 0.5%, 1%, 2.5%, 5%, and 10% tails of the return distribution). The last row gives the likelihood ratio (LR) statistics of the unconditional coverage test and conditional coverage test. *** denote significance at the 1% levels.

**Table A6:** Out-of-sample conditional VaR performance of GARCH-type models with standardized residuals following Johnson’s SU distribution for WTI crude oil returns.

Θ	GARCH	EGARCH	NAGARCH	AGARCH	GJR-GARCH	IGARCH	TGARCH	TS-GARCH
0.5%	−3.9160	−3.7803	−3.8760	−3.8989	−3.9246	−3.9523	−3.8061	−3.8442
N (0.5%)	28(29)	21(29)	16(29)	26(29)	43(29)	57(29)	15(29)	19(29)
LR^uc^/LR^cc^	0.08/2.43	2.72 */6.14 **	7.44 ***/7.52 **	0.43/3.05	5.46 **/19.11 ***	20.25 ***/39.10 ***	8.73 ***/8.80 **	4.29 **/4.41 ***
1%	−3.0520	−2.9421	−3.0202	−3.0389	−3.0402	−3.0821	−2.9663	−2.9949
N (1%)	58(58)	60(58)	35(58)	54(58)	81(58)	113(58)	39(58)	42(58)
LR^uc^/LR^cc^	0.02/29.42 ***	0.02/22.82 ***	11.51 ***/13.10 ***	0.43/20.55 ***	7.45 ***/40.65 ***	39.44 ***/66.58 ***	7.75 ***/12.62 ***	5.48 **/19.50 ***
2.5%	−2.1142	−2.0303	−2.0897	−2.1045	−2.1059	−2.1359	−2.0504	−2.0170
N (2.5%)	159(147)	155(147)	94(147)	142(147)	219(147)	307(147)	104(147)	110(147)
LR^uc^/LR^cc^	0.91/21.47 ***	0.40/25.42 ***	22.72 ***/58.89 ***	0.21/26.80 ***	31.11 ***/56.33 ***	135.76 ***/167.51 ***	14.58 ***/45.72 ***	10.65 ***/39.07 ***
5%	−1.5362	−1.4668	−1.5151	−1.5279	−1.5283	−1.5518	−1.4829	−1.5002
N (5%)	372(294)	358(294)	242(294)	328(294)	499(294)	611(294)	246(294)	247(294)
LR^uc^/LR^cc^	19.72 ***/48.43 ***	13.39 ***/43.19 ***	10.57 ***/40.40 ***	3.80 */34.28 ***	124.39 ***/147.18 ***	276.39 ***/307.46 ***	8.99 ***/42.51 ***	8.61 ***/39.12 ***
10%	−1.0509	−0.9922	−1.0318	−1.0434	−1.0421	−1.0608	−1.0040	−1.0197
N (10%)	766(589)	758(589)	571(589)	703(589)	950(589)	1104(589)	600(589)	585(589)
LR^uc^/LR^cc^	54.11 ***/81.25 ***	49.48 ***/71.81 ***	0.66/99.33 ***	22.97 ***/49.86 ***	210.58 ***/224.63 ***	407.73 ***/420.33 ***	0.20/25.77 ***	0.03/27.96 ***

Notes. This table shows statistics on the out-of-sample performance of eight variations of the GARCH model with Johnson’s SU distribution. The skewness and kurtosis parameters are defined as a function of the innovation at period t−1 and autoregressive of order 1. The results are based on the daily returns of the WTI crude oil price from 4 January 1988 to 30 June 2021 (8439 observations). A 10-year rolling sample, starting from 4 January 1988, is used to estimate conditional VaR measures, and a 1-year holdout sample is used to evaluate their performance. The values in the first row in each panel are the average estimated thresholds. The values in the second row present the actual counts and the expected counts in parentheses (i.e., the number of observations falling in the 0.5%, 1%, 2.5%, 5%, and 10% tails of the return distribution). The last row gives the likelihood ratio (LR) statistics of the unconditional coverage test and conditional coverage test. *, **, and *** denote significance at the 10%, 5%, and 1% levels, respectively.

**Table A7:** Out-of-sample conditional VaR performance of GARCH-type models with standardized residuals following Johnson’s SU distribution for Brent crude oil returns.

Θ	GARCH	EGARCH	NAGARCH	AGARCH	GJR-GARCH	IGARCH	TGARCH	TS-GARCH
0.5%	−4.0481	−3.8576	−4.0076	−4.0721	−3.9415	−3.9948	−3.8213	−3.9438
N (0.5%)	19(29)	15(29)	13(29)	15(29)	30(29)	39(29)	12(29)	13(29)
LR^uc^/LR^cc^	4.52 **/23.03 ***	9.05 ***/30.64 ***	12.59 ***/32.60 ***	9.05 ***/30.64 ***	0.00/26.98 ***	2.59/24.07 ***	18.83 ***/38.43 ***	12.09 ***/35.60 ***
1%	−3.1574	−3.0062	−3.1274	−3.1784	−3.0756	−3.1170	−2.9789	−3.0758
N (1%)	46(59)	37(59)	22(59)	34(59)	59(59)	77(59)	23(59)	26(59)
LR^uc^/LR^cc^	3.41 */21.56 ***	10.02 ***/32.59 ***	31.61 ***/56.45 ***	13.16 ***/37.49 ***	0.01/23.42 ***	4.68 **/20.97 ***	29.65 ***/45.73 ***	24.28 ***/54.34 ***
2.5%	−2.1923	−2.0582	−2.1712	−2.2092	−2.1330	−2.1626	−2.0601	−2.1325
N (2.5%)	128(149)	99(149)	67(149)	98(149)	187(149)	255(149)	78(149)	79(149)
LR^uc^/LR^cc^	3.20 */21.49 ***	19.51 ***/36.55 ***	58.11 ***/83.08 ***	20.36 ***/47.75 ***	9.18 ***/18.02 ***	63.90 ***/77.03 ***	41.94 ***/62.24 ***	40.63 ***/60.55 ***
5%	−1.5989	−1.5126	−1.5816	−1.6125	−1.5505	−1.5735	−1.4909	−1.5504
N (5%)	357(298)	307(298)	177(298)	291(298)	487(298)	578(298)	215(298)	211(298)
LR^uc^/LR^cc^	11.55 ***/36.62 ***	0.27/10.33 ***	60.23 ***/73.36 ***	0.17/12.02 ***	106.64 ***/124.30 ***	219.71 ***/234.99 ***	26.89 ***/41.80 ***	29.70 ***/45.64 ***
10%	−1.1019	−1.0310	−1.0867	−1.1126	−1.0605	−1.0786	−1.0106	−1.0614
N (10%)	746(596)	691(596)	515(596)	660(596)	944(596)	1096(596)	605(596)	578(596)
LR^uc^/LR^cc^	39.05 ***/58.20 ***	16.02 ***/29.17 ***	12.82 ***/29.36 ***	7.36 ***/21.51 ***	195.08 ***/207.07 ***	345.72 ***/354.03 ***	0.14/16.01 ***	0.62/13.89 ***

Notes. This table shows statistics on the out-of-sample performance of eight variations of the GARCH model with Johnson’s SU distribution. The skewness and kurtosis parameters are defined as a function of the innovation at period t−1 and autoregressive of order 1. The results are based on the daily returns of the Brent crude oil price from 4 January 1988 to 30 June 2021 (8500 observations). A 10-year rolling sample, starting from 4 January 1988, is used to estimate conditional VaR measures, and a 1-year holdout sample is used to evaluate their performance. The values in the first row in each panel are the average estimated thresholds. The values in the second row present the actual counts and the expected counts in parentheses (i.e., the number of observations falling in the 0.5%, 1%, 2.5%, 5%, and 10% tails of the return distribution). The last row gives the likelihood ratio (LR) statistics of the unconditional coverage test and conditional coverage test. *, **, and *** denote significance at the 10%, 5%, and 1% levels, respectively.
